# Longitudinal analysis of human milk oligosaccharides and mucin-2-glycans in infants reveals enrichment of sulfated and glucuronic acid-bearing HMOs

**DOI:** 10.1016/j.jbc.2026.113099

**Published:** 2026-04-28

**Authors:** Nitin, Parandis Daneshgar, Darrek Kniffen, Sara D. Vicaretti, Brandon Whitmore, Linus Kipchumba, Candice Quin, Deanna L. Gibson, Kirk S.B. Bergstrom, Wesley F. Zandberg

**Affiliations:** 1 Department of Chemistry, The University of British Columbia, Kelowna, British Columbia, Canada; 2 Department of Biochemistry and Molecular Biology, The University of British Columbia, Kelowna, British Columbia, Canada; 3 Department of Biology, The University of British Columbia, Kelowna, British Columbia, Canada

**Keywords:** human milk oligosaccharides (HMOs), mucus, *O*-glycans, mass spectrometry, capillary electrophoresis, metabolism, glycomics, stool, glucuronic acid, sulfate

## Abstract

In infants, the acquisition of an adult-like gastrointestinal (GI) microbiome begins at birth and fully develops by toddlerhood. This colonisation is influenced by the diet, which, in breast-fed infants, contains high concentrations of soluble human milk oligosaccharides (HMOs), prebiotics that bear structural resemblance to the glycans that decorate Mucin-2 (MUC2). MUC2 is the first line of defense between the microbiota and the underlying mucosal tissues; it also provides a nutrient-rich habitat for GI commensal microbes. The research described herein builds on two earlier findings: the ability to sample MUC2 adhering to the surface of stool samples and the discovery of a class of sulfated HMOs within human milk. Studies have shown that de-sulfation of MUC2-borne glycans is a key to their metabolism by GI microbes *in vitro* and, if sulfated glycans are otherwise metabolically-resistant, two hypotheses follow. First, in contrast with major prebiotic HMOs, sulfated HMOs will be enriched in the infant GI tract; second, high abundances of glyco-epitopes like sulfation in HMOs will deflect the microbiome’s metabolic activity away from MUC2, leading to detectable changes in the host glycome. We show that both HMOs and MUC2 could be non-invasively sampled from the same infant stool samples. Capillary electrophoresis analyses of paired milk/stool samples demonstrated significant enrichment of sulfate-containing HMOs in stool. High-resolution mass spectrometry (HRMS) was used to detect numerous sulfated HMOs, including a new class concurrently containing glucuronic acid (GlcA) residues. Infant MUC2-derived glycans were also analyzed by HRMS; this is the first reported semi-quantitative longitudinal study and provides a benchmark for future research.

Mucus, a gel-like substance secreted by the goblet cells of the gastrointestinal (GI) tract, fills numerous health-promoting roles, such as lubrication, host-defense against pathogens and toxins, and promoting anti-inflammatory actions against the GI microbiota (1). These diverse roles are facilitated by the unique molecular features of mucus, in particular the gel-forming protein Mucin-2 (MUC2; Muc2 in mice). MUC2 is highly glycosylated, with almost two thousand oligosaccharides glycosidically bound to the hydroxyl group of serine (Ser) or threonine (Thr) residues (*i.e.*, *O*-glycans). Over 200 unique *O*-glycan structures have been identified on MUC2 that together constitute up to 80 percent of its enormous 2 MDa mass (1, 2). Clearly defining the factors that influence the composition of the MUC2 glycome is necessary for understanding its contributions to GI health and disease. Complex and diverse MUC2 glycans typically are composed of one to 10 monosaccharides glycosidically linked to a common Ser/Thr-linked *N*-acetyl-D-galactosamine (GalNAc) residue (Fig. S1*A*). Glycan diversity is achieved by the addition of up to five different monosaccharides (like GalNAc, *N*-acetyl-D-glucosamine (GlcNAc), D-galactose (Gal), L-fucose (Fuc), and *N*-acetyl-D-neuraminic acid (Neu5Ac, also known as sialic acid) by competitive glycosyltransferase (GTs) that frequently yield branched structures. Eight different *O*-glycan core structures varying in the monosaccharide combinations appended directly to the reducing end GalNAc residue have been described, six of which have been detected on human MUC2 (3, 4, 5, 6, 7, 8, 9) (Fig. S1*B*). Further glycan diversity is established by sulfotransferases that decorate GlcNAc and Gal residues with sulfate moieties (7, 8, 9), as well as the *O*-acetylation of Neu5Ac hydroxyl groups (7). In mice, two functionally distinct layers of Muc2 protect the colon (10), the site of the highest concentration of microbes in the GI tract: a diffuse “niche” Muc2 layer that is actively colonized by microbes, many of which possess the capacity to metabolize Muc2-borne glycans to meet their metabolic needs, and a denser “barrier” layer that is normally devoid of microbes. The glycans borne by these two Muc2 layers are distinct and studies in mice have shown that alterations in *O*-glycan core structures spontaneously leads to microbiota-triggered chronic inflammation associated with disease outcome in rodents (1, 11). Detailed analyses of the *O*-glycome of the adult human GI tract (7, 8) have revealed mostly core-3-based extended or branched glycans (frequently α2,6 sialylated on the initiating GalNAc) and the presence of an acidic (sulfate or Neu5Ac) gradient increasing from the ileum to colon. This tissue-specific change in the glycomic environment has been hypothesized to be linked to the specific microbes that inhabit different regions of the GI tract (7). As in mice, glycomic changes have been observed in the GI MUC2 of patients with active ulcerative colitis (12). Fetal (or neonatal) MUC2, although much less studied than adult tissues, is thought to contain mostly core-2 type glycans (9), an observation that, should it be supported by further evidence, would lead to two, non-mutually-exclusive hypotheses: first, that an infant (core-2) *versus* adult (core-3) MUC2 glycan class-switch is associated with the acquisition of an adult-like GI microbiome, and second, that the infant diet (*i.e*. milk *vs*. solid foods) is linked to these alterations to the MUC2 glycome. Intriguingly, one component of the infant diet is notably absent from that of adults’ bears striking chemical resemblance to MUC2: human milk oligosaccharides (HMOs; Fig. S1*C*).

Microbial colonization of the infant GI tract begins at birth, quickly progressing towards a community dominated by species able to metabolize prebiotic HMOs that reach the distal GI tract undigested in contrast to lactose from which they are biosynthesized (13, 14, 15). Some GI bacteria that achieve persistence in the infant gut within the first year of life, *e.g. Bacteroides thetaiotaomicron* and *Bacteroides fragilis*, are able to meet their energy needs by metabolizing HMOs *via* identical processes used to access MUC2 glycans (16). Colonization of the GI tract by these HMO-consuming microbes has been associated with altered profiles of health-promoting metabolites *in vitro* (17) and *in vivo* (18). A recent study has demonstrated that an HMO concentrated from pooled milk also altered the microbiome of healthy adults in a dose-dependent manner; interestingly, this microbial influence could not be replicated with defined mixtures of the 10 most abundant HMOs suggesting that even low abundance HMOs may functionally affect the GI tract (19). These prebiotic activities are structure-specific and to date, over 200 unique HMOs have been identified. Like MUC2-borne glycans, HMOs are produced by a range of competitive GTs. Genetic factors regulating the expression of GTs, especially fucosyltransferase (FUT) two and FUT3, are major sources of HMO variation between women. FUT2 catalyzes the transfer of α1,2-Fuc residues to HMOs and women lacking this enzyme—roughly 30 percent of the world’s population—are known as secretor negative (Se−). Secretor-positive (Se+) women produce milk rich in the HMOs 2′fucosyllactose (2′FL) and lacto-N-fucopentaose 1 (LNFP1), while only traces of these are present in Se-milk (20). Expression of active FUT3 results in the biosynthesis of HMOs bearing Lewis (Le) antigens that contain α1,3 or α1,4-Fuc residues; 3-fucosyllactose (3FL) and LNFP2 are HMOs that characterize Le+ milk. In addition to genetics and lactation stage (*i.e.*, days *postpartum*), other environmental and maternal factors (21) are also known to influence HMO biosynthesis; for example, geographical variance (20) in HMO concentration has been observed, while we (22) and others (23) have detected associations between the diet and HMOs. The plethora of factors influencing the HMO glycome are important as both their abundance and diversity has clearly been associated with altered infant GI microbiomes (14, 23) and consequent health outcomes (24, 25).

In addition to their role in shaping the infant GI microbiome *via* prebiotic mechanisms, HMOs, by mimicking MUC2 glycans, are thought to possess anti-infective functions limiting pathogen adherence (24) to the GI mucosa. Though mechanistic details in humans remain incomplete, many hypothesis-generating studies have documented inverse-associations between concentrations of specific HMOs and rates of bacterial or viral disease (25) and *in vitro* and animal experiments have further supported their anti-pathogenic effects. Evidence of HMOs in the plasma (26) and urine (27) of breastfed infants also suggests that they may prevent infections beyond the GI tract and indicates their potential to modulate immune cell activity both locally, in the GI-associated lymphoid tissues, as well as more distal sites in the body (28). For example, at least 25 different HMOs have been shown to bind to galectins, GI epithelial- and T-cell-surface proteins that bind lactose- or *N*-acetyllactosamine-containing glycans to mediate immune responses, inflammation, and cell-signaling (29). Selectins, cell-adhesion molecules responsible for initiating the early stages of leukocyte trafficking, are another class of lectins that are able to bind HMO-borne Le epitopes (30). Specifically, the cytokine-induced expression of P- and E-selectin on endothelial cells enables them to bind to leukocyte-expressed Le antigens facilitating their extravasation and infiltration into the mucosa. Finally, sialic acid-binding immunoglobulin-type lectins (Siglecs) have been shown to interact with some milk oligosaccharides (MOs) (31) a finding that may be especially relevant for the developing infant immune system since the activity of immune cell-borne Siglecs prevents inappropriate responses against self-antigens. Our recent reports of a previously under-characterized class of sulfated MOs in both bovine and human milk is significant with respect to their immune functions for two reasons. First, 3′-sulfo-lactose (3′SO_3_L) has been shown to bind to both galectins and selectins, an activity that would directly antagonize their engagement with ligands on immune cells; note that sulfation also fine-tunes the recognition of Siglec ligands (32). Second, sulfation has been shown to directly impede the microbial metabolism of MUC2 glycans (33, 34) and thus it is predicted that sulfation would have a similar impact on HMO metabolism which would consequently enhance their persistence, promoting immune functions in the GI tract and circulation.

Recently, based on high-resolution mass spectrometry (HRMS) evidence, we reported the existence of putative MOs in bovine milk bearing both sulfate and uronic acid (presumably D-glucuronic acid; GlcA) residues (35). Prior to this report (35), GlcA had not been identified as a constituent of MOs in any species. Glycans bearing sulfated GlcA are key features of the CD57 epitope (also called HNK1), a marker of terminally differentiated natural killer (NK) cells, suggesting that these unusual MOs may possess immune-regulatory properties. Jin and colleagues have expanded the list of mammals in which GlcA residues have been discovered in MOs, using MS to detect these monosaccharides in MOs isolated from the milk of non-domestic mammals (36). These researchers also demonstrated that 3′-glucuronyl-lactose (GlcA-L) potently attenuated the lipopolysaccharide (LPS)-induced production of inflammatory cytokines by human macrophages. To extend the relevance of these observations to human infant nutrition, we sought to assess whether sulfate- and GlcA-containing HMOs could similarly be detected in human milk. Additionally, we sought to test if sulfated HMOs, whether bearing GlcA or not, were uniquely enriched in stool samples collected from exclusively breastfed infants; such enrichment, which would substantiate the potential immune *vs.* prebiotic functions of these HMOs, is anticipated given the metabolic-recalcitrance of structurally-similar sulfated MUC2 glycans. Finally, we sought to uncover associations between the milk and MUC2 glycome in exclusively breastmilk-fed infants, testing, for example, whether higher relative abundances of fucosylated or sialylated HMOs deflected microbial fucosidase- or sialidase-activity away from MUC2-bound glycans leading to a consequent elevation of these residues in mucus. It has recently been shown by us (37) and others (38) that MUC2 can be effectively purified from a stool sample for glycomic analyses. In the present study, our fecal MUC2 extraction procedure was applied to a longitudinal set of infant stool samples collected from four mother/infant pairs (22, 39). Breastmilk was the sole source of nourishment for these infants, and since milk and stool collection took place on the same day, the extraction and semi-quantitation of HMOs from each sample permitted the assessment of whether sulfated HMOs were metabolically resistant (as hypothesized) and whether their abundances influenced the neonatal MUC2 glycome. Using capillary electrophoresis with laser-induced fluorescence detection (CE-LIF) and high-performance liquid chromatography (HPLC) with HRMS detection, we demonstrate that sulfate-bearing HMOs are uniquely enriched within the infant GI tract. Further, we provide HRMS evidence for a previously undescribed class of HMO bearing both GlcA and sulfate moieties. Finally, we provide the first (to the best of our knowledge) in-depth, semi-quantitative analysis of the neonatal MUC2 glycome, demonstrating that core-2 glycans appear to be inversely correlated with more adult-like core-3 glycans and permitting a study associating relative abundances of HMOs to alterations in the MUC2-borne *O*-glycans.

## Results

### Evidence for sulfated and GlcA-containing HMOs in breastmilk

**Figure 1:**
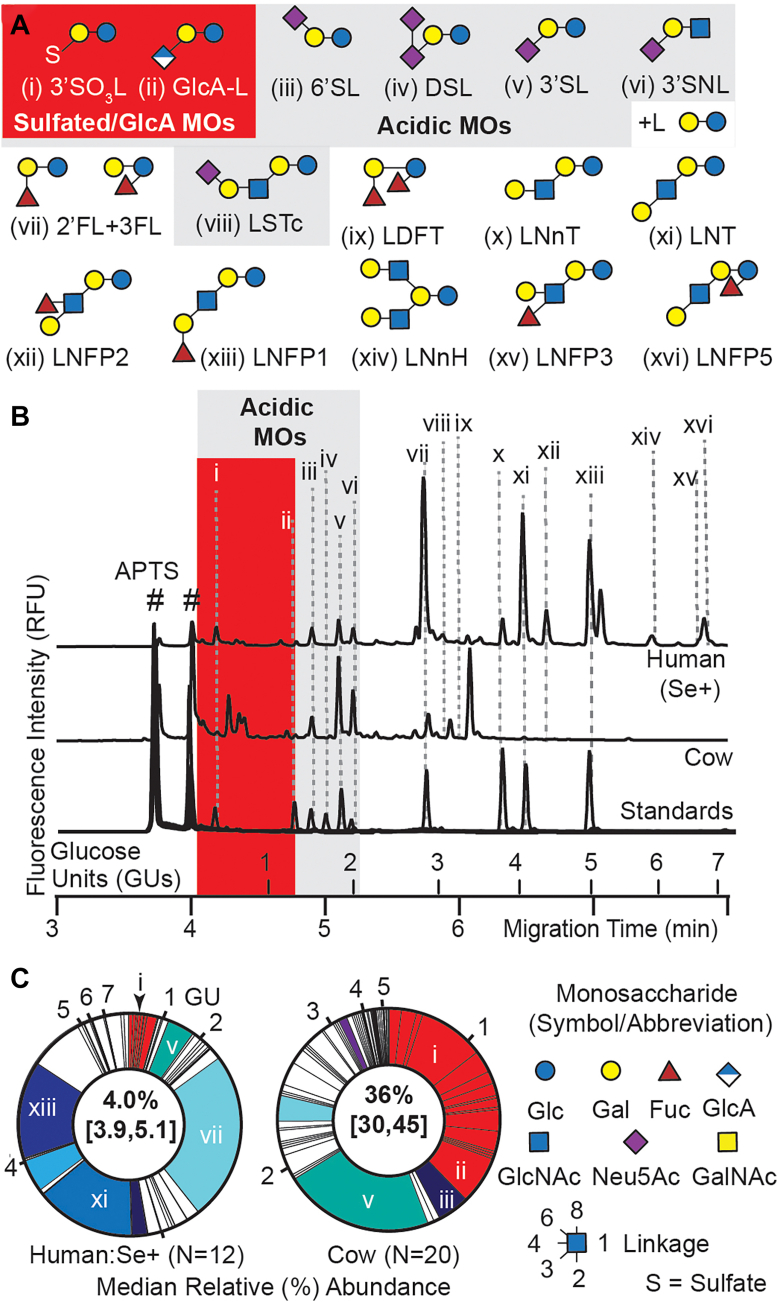
**Electrophoretic evidence for the existence of a large class of non-sialylated yet acidic HMOs.***A*, HMO standards used for annotating CE-LIF and HPLC-MS data. All acidic HMOs are outlined in gray while the subset lacking Neu5Ac residues is outlined in *red*. Note that CE-LIF is unable to resolve 2′FL and 3FL or 3′SNL and lactose (L); accordingly, 2′FL/3FL are reported together, and the 3′SNL/L peak was not included in the total area used to determine relative abundances of the other HMOs. *B*, representative CE-LIF electropherograms from mature human (Se+) and bovine milk aligned with selected HMO standards demonstrate the existence of a class of HMOs (shaded in *red*) that exceed the mobility of 6′SL, the fastest eluting, sialylated HMO; these highly mobile HMOs are hypothesized to contain GlcA and/or bear one or more sulfated monosaccharide residue(s). Peaks labelled with the # sign are attributable to the APTS labelling reagents. *C*, median relative abundances of putative sulfate- and/or GlcA-containing milk oligosaccharides (MOs) in cow and human milk as assessed by CE-LIF; values for first and third quartiles are indicated in square brackets. Each wedge of the pie graph denotes a different MO, beginning with the first peak not attributable to the fluorogenic labelling reagents; all peaks are listed in order of CE migration time. Lower case Roman numerals refer to HMO standards as depicted in Figure 1*A*. Wedges shown in white denote HMOs for which standards were not available while those in red represent the *red* highlighted region of electropherograms as depicted in Figure 1*B*. The Arabic numerals on the outside of each pie graph denote the position of a starch hydrolysate-derived mobility marker of the same number of glucose units (GU) and corresponds to the GUs in the x-axis of Figure 1*B*. The monosaccharide symbols are used as recommended by the Consortium for Functional Glycomics (75).

**Figure 2:**
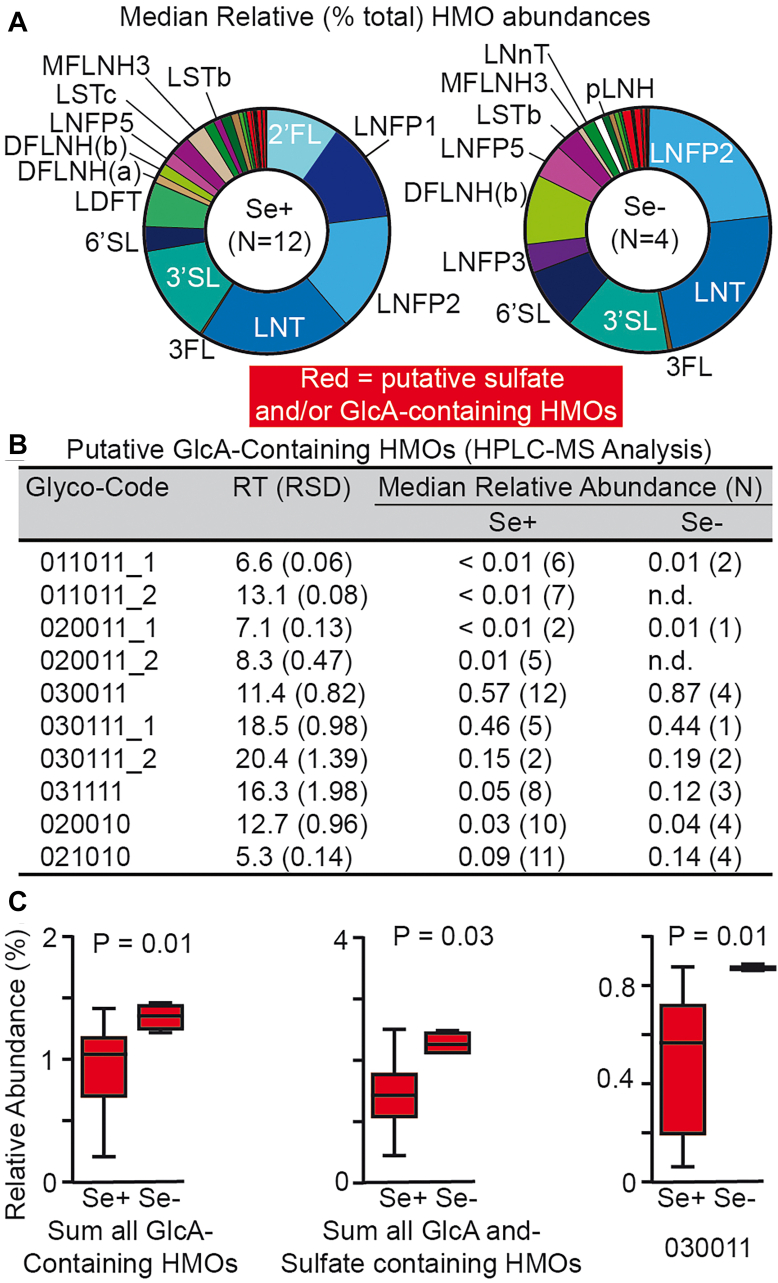
**Full-scan HPLC-MS analysis revealed putative GlcA-containing and sulfated analogues in human breastmilk.***A*, Median relative (%) abundances of 22 known HMOs and 10 HMOs with formulas consistent with GlcA and/or sulfate residues in milk collected 5-months *postpartum* from Se+ (N = 12) and Se- (N = 4) donors. Wedges of the pie graphs are color-coded exactly as in Figure 1*C* with all sulfate/GlcA-containing HMOs in *red*. *B*, list of all GlcA-containing HMOs detected and median relative abundances (*i.e*., % of total HMOs) for Se+ and Se-donors. Note that each glycan is labelled with a unique seven-digit glyco-code with the first six digits sequentially specifying the number of *m*/*z*-resolvable *N*-acetylhexosamine (HexNAc), hexose (Hex), Fuc, Neu5Ac, GlcA, and sulfate residues and the _1, _2 suffixes referring to the number of HPLC-resolvable isobars. n.d. = not detected. Mean retention times (RT) in minutes are reported with relative standard deviations (RSD) listed in parentheses. *C*, relative abundances of total GlcA-containing HMOs and 030,011 were significantly higher in Se-milk samples than Se + ones (*p* = 0.03 and 0.01, respectively; unpaired, Mann–Whitney *U* test). Boxplot center lines show medians and limits indicate 25th and 75th percentiles; whiskers extend to 1.5 times the interquartile range from the 25th and 75th percentiles.

We have previously used CE-LIF to profile the MOs in both bovine (35, 40) and human milk (22). CE provides a rapid method to resolve glycans based on both their size and charge while LIF detection simplifies the establishment of quantitative relationships between HMOs within samples since identical detector responses are achieved by the shared fluorophore (41, 42). Acquisition of two HMO standards—GlcA-L and disialyllactose (DSL; Fig. 1*A*)—that were not available in our previous studies permitted the unambiguous identification of a region within CE-LIF electropherograms (shaded red in Fig. 1*B*) wherein HMO mobility exceeded that of the fastest known HMO in which the negative charge was localized on a Neu5Ac residue (6′SL); we hypothesized that these exceptionally highly mobility HMOs contained charge-carriers smaller than Neu5Ac, namely sulfates, GlcA residues, or combinations of both. Relative abundances of these acidic, putatively sulfated or glucuronylated HMOs were appreciable, with total median concentrations in mature Se + human milk of 4%, and reaching 36% in bovine milk (Fig. 1*C*). Note that Se+ and Se-donors were defined based on the high relative concentrations of 2′FL, LDFT, and LNFP1 in the former and their near absence in the latter (13, 22, 41, 42). To define putative GlcA/sulfate-containing HMOs in breastmilk with greater chemical clarity, an HPLC-MS data set previously obtained from a cross sectional set of 16 milk samples (N = 12 Se+ and 4 Se-) collected 5-months *postpartum* (22) was mined for chemical formulas (*i.e*. *m*/*z* ratios) consistent with GlcA-containing HMOs (Fig. 2). Ions consistent with the *m*/*z* ratios of 10 putative GlcA-containing HMOs were detected (Fig. 2*B*) and relative abundances of these with respect to a set of HMOs identified using commercial standards as well as previously identified putative sulfated HMOs were determined (Figs. 1*A* and 2; Table S1). Median relative abundances of all GlcA-containing HMOs for Se+ (1.07%) and Se- (1.39%) samples were significantly different (*p* = 0.01) consistent with the observation that the most abundant HMO in this class (030011) likewise significantly differed (*p* = 0.01) between these groups (Fig. 2*C*). As was previously observed in bovine milk (35), most (eight out of ten) GlcA-containing HMOs also bore sulfate residues. Collectively, median concentrations of HMOs bearing any one of either sulfate or GlcA residues were significantly (*p* = 0.03) higher in Se-samples (2.90%) than Se + samples (2.06%). Interestingly, several putative HMOs possessed Neu5Ac, GlcA, and sulfate groups, an unusual tri-anionic glycan-epitope that, to our knowledge, has not been previously reported in HMOs. We explored this through structural studies as described below.

### Sulfate/GlcA-containing HMOs: Stability during lactation and enrichment in neonatal GI tract

**Figure 3:**
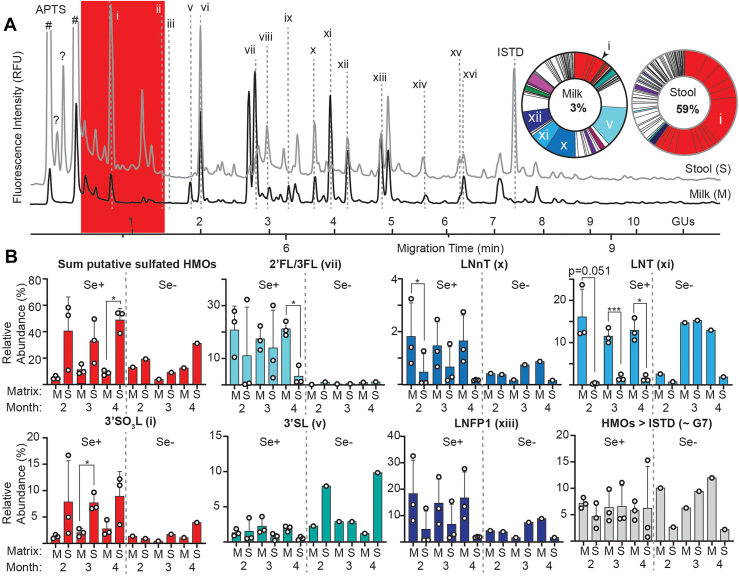
**CE-LIF analysis indicated that sulfated HMOs were enriched in stool *vs.* milk.***A*, representative CE-LIF electropherograms depicting HMO profiles in milk before (*black*) and after (*gray*) digestion. All peaks not attributable to the APTS derivatizing reagents, which are labelled with the # sign, were sequentially integrated and their relative areas are depicted in the pie charts in the order in which they migrate with wedges color-coded exactly as in Figures 1 and 2. Relative abundances of 16 HMOs that were identified with standards (structures corresponding to each lowercase Roman numeral are provided in Figure 1 and Fig. S2) were compared within all milk/stool samples. Additionally, peak areas for two larger regions of each electropherogram were determined in aggregate: the highly mobile region predicted to contain the majority of sulfated and/or GlcA-containing HMOs (highlighted in *red*) as well as a low-mobility region eluting after the maltoheptaose ISTD. *B*, mean relative abundances of selected HMOs or HMO classes in milk and stool. Error bars denote standard deviation. Significant differences in relative HMO abundance, for Se+ samples only, were evaluated using a two-way, paired *t* test with ∗, ∗∗, and ∗∗∗ indicating *p* < 0.05, 0.01, and 0.005, respectively.

In order to test the hypothesis that sulfate-containing HMOs were resistant to metabolism within the infant GI tract, and also to better characterize novel GlcA-containing HMOs, a longitudinal set of paired milk/stool samples was acquired from four exclusively breastfed infants. Maternal secretor-status (N = 3 Se+, and 1 Se-) was again deduced on the basis of relative 2′FL, LDFT, and LNFP1 concentrations; however, the infants’ secretor status was unknown. Milk, as well as water-soluble material within the stool samples (*i.e*. HMOs that survived digestion), were subjected to identical HMO extraction procedures before fluorescence derivatization and analysis by CE-LIF. Since it was not possible to determine how much milk each infant ingested, relative rather than absolute abundances of HMOs in milk and stool were evaluated (Fig. 3). CE-LIF analysis revealed that HMO-like peaks could be recovered from stool samples, many of which co-migrated with standards (Fig. 3*A*). While some HMOs appear to be unambiguously metabolized, *e.g.* 2′FL/3FL (vii), LNnT (x) or LNT (xi), others, in particular 3′SO_3_L (i) and most of the other putative sulfated HMOs (shaded in red), were highly enriched, for example rising from 3% of the total HMOs detected in milk to 59% in the paired stool sample shown (Fig. 3*A*). Two peaks, labelled “?”, had mobilities close to those associated with the APTS labeling reagents but were neither detectible in milk nor control samples, containing only the APTS labelling reagents; given their extremely high mobilities, we hypothesize that these may be the HMOs bearing two or more negative charges, *e.g*. GlcA or Neu5Ac plus one or more sulfate group(s), that were detectible in milk by HPLC-MS (Fig. 2*B*). Inspection of electropherograms also indicated numerous peaks in stool samples with mobilities exceeding that of the maltoheptaose (an oligomer of seven glucose (G) residues) internal standard (ISTD). Although these later migrating peaks were more difficult to align and reproducibly integrate (which is why peak areas in this region of electropherograms were determined in aggregate), stool samples frequently possessed peaks in this low-mobility region that were not readily apparent in corresponding milk samples; these low mobility peaks may reflect the release of large *N*-glycans from milk proteins as previously noted by Davis *et al.* (43). Analysis of the relative abundance of selected HMOs or groups of HMOs revealed no obvious longitudinal trends or differences between individual donors besides Se-status (Fig. S3) and accordingly mean abundances (for Se + samples only) in milk and stool were compared at 2-, 3-, and 4-months *postpartum* (Fig. 3*B*). Among the Se+ mother/infant pairs, several HMOs clearly exhibited prebiotic trends in that they exhibited substantial reductions in their relative abundance in stool relative to milk; these prebiotic HMOs included LNT (xi), LNnT (x), and, less frequently, 2′FL/3FL (vii) and LNFP1 (xiii). Some HMOs, particularly those bearing Neu5Ac residues or with mobilities slower than the ISTD, appeared to maintain equivalent relative concentrations before and after digestion, although 3′SL (v; Fig. 3*B*) and LSTc (viii; Fig. S3) were concentrated in several individual stool samples. Among Se+ samples, relative abundances of total, putative sulfated HMOs, as well as 3′SO_3_L (i) specifically, were enriched in all stool samples relative to their paired milk samples, consistent with the hypothesis that sulfation impedes microbial-catalyzed metabolism of HMOs or glycans.

### Novel GlcA-containing HMOs detected by HPLC-MS in milk and stool

**Figure 4:**
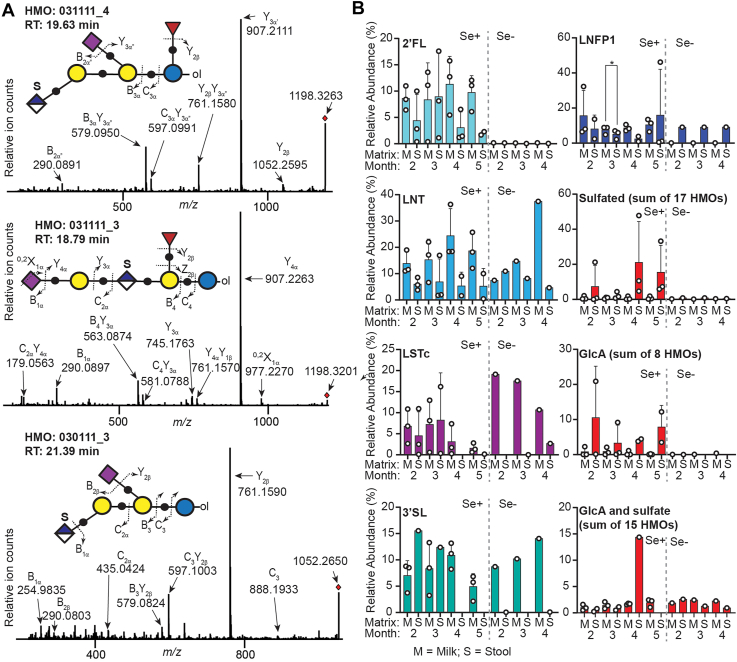
**HPLC-MS evidence for a large class of sulfate- and GlcA-containing HMOs that were also recovered from stool samples from exclusively breastfed infants.***A*, collisional-induced dissociation (CID) tandem mass spectra for three sulfate-, GlcA-, and Neu5Ac-containing HMOs. The nomenclature of Domon and Costello (74) is used to describe the product ions for each [M-H]^-^ precursor ion; [M-H]^-^ ions in each spectrum are labelled with the small, red diamond. The inter-glycosidic oxygen atoms for HMOs are denoted by the small *black circles* while all monosaccharide and sulfate residues are defined as in Figure 1*C*. *B*, mean relative abundances of selected HMOs or HMO classes in milk and stool. Error bars denote standard deviation. Significant differences in relative HMO abundance, for Se+ samples only, were evaluated using a two-way, paired *t* test with ∗ indicating *p* < 0.05. Bars are color-coded exactly as in Figure 1, Figure 2, Figure 3.

Analysis of milk and stool HMO abundances by HPLC-HRMS (Fig. 4) afforded evidence for a large class of GlcA- and/or sulfate-containing HMOs (Table S2). Including GlcA-L, full scan mass spectral data for a total of 40 putative HMOs were acquired; 17 of these HMOs were sulfated only, eight bore only GlcA-residues, and 15 HMOs contained both GlcA and sulfate moieties. Consistent with the cross-sectional data set (Fig. 2*B*) seven HMOs with mass-to-charge ratios reflecting the simultaneous presence of GlcA, Neu5Ac, and sulfate residues were detected (Fig. S4); three of these were of sufficient abundance to permit the collection of high-resolution product ion spectra (Fig. 4*A*). In all three instances, the base peak in the product ion spectra was a Y-ion resulting from the loss of a Neu5Ac residue, an observation that supports the presence of the Neu5Ac/Fuc pair over the isobaric combination of Neu5Gc and Gal for the fucosylated HMOs 031111_4 and 031111_3. HMOs 030111_3 and 031111_4 both appeared to be branched, with the sulfated GlcA residues borne on a non-reducing Hex (presumably Gal); meanwhile, the *m*/*z* 907 (Y_4α_) and 745 (Y_3α_) pair of ions in 031111_3—which is consistent with the neutral loss of a Hex residue—suggested that this HMO is linear with an internal sulfated GlcA moiety. A prominent pair of non-fucosylated B_3α_Y_3α”_ and C_3α_Y_3α”_ ions for 031111_4 indicated that the fucose residue for this HMO was on the reducing end, *i.e.* this HMO is likely based on a 3FL core; in contrast, the complementary B_4_Y_3α_ and C_4_Y_3α_ pair for the isobaric 031111_3 ion indicated the presence of a non-reducing fucose residue, suggestive that this HMO was elaborated off of a 2′FL core. Additional tandem MS spectra for four previously undescribed sulfated HMOs are also presented in Fig. S5; these data support sulfation of both Gal and GlcNAc residues. As was observed by CE-LIF, longitudinal analysis of all samples exhibited no clear trends with respect to changes in relative HMO abundances as a function of time *postpartum* (Fig. S6) and accordingly monthly data for Se+ samples were again averaged to permit a comparison of relative abundances in milk *versus* paired stool samples (Fig. 4*B*). 2′FL and LNT again appeared to be the major prebiotic HMOs and, although their relative abundances where quite variable, they were present at lower relative abundance in stool samples compared to milk. Conversely, while not always consistently detected by HPLC-HRMS, the abundances of sulfate and GlcA-containing HMOs present in stool samples were equal to or greater than those present in matched milk samples.

### Glycomic analysis of neonatal GI MUC2

Early colonizers of the infant GI tract like *Akkermansia muciniphila* (44, 45) and *Bacteroides thetaiotomicron* (16) can metabolize both HMO and MUC2-derived glycans *via* similar pathways. A study in which infants were inoculated with *Bifidobacterium longum* subsp. *infantis* reported that significantly lower concentrations of HMOs were recoverable from stool in the treated *versus* the control group (46) while concurrently lower relative abundances of free *O*-glycans (47) were also observed in the inoculated infants. Presumably, these *O*-glycans originated from the endo-*O*-glycanase-catalyzed metabolism of MUC2-bound glycans (48). Collectively, all of these studies suggest that microbial foraging of HMOs may deflect comparable activity away from the MUC2-borne glycans; however, to our knowledge, this hypothesis has not yet been directly assessed *in vivo*. Accordingly, in a proof-of-concept and hypothesis-generating test, we evaluated if an extraction protocol initially developed for isolating MUC2 that normally adheres to the surface of a stool sample (10, 37) could be extended to sub-optimally collected samples, namely, stool samples obtained from soiled diapers and homogenized in preparation for metagenomic microbiome analyses. Beginning with between 5 and 110 mg of freeze-dried stool, minor adjustments to the MUC2 extraction procedure (see Experimental procedures) yielded high-purity, high-molecular-weight glycoproteins when assessed by composite sodium dodecyl-sulfate-urea-agarose-polyacrylamide gel electrophoresis; these glycoproteins positively reacted with an anti-human MUC2 antibody as well as lectins known to recognize MUC2-borne glycans (Fig. S7). After cleaving *O*-glycans from the MUC2 polypeptide backbone using well-established reductive β-elimination protocols (49), subsequent HPLC-HRMS analyses afforded evidence for 160 unique putative *O*-glycans across 15 separate samples. These *O*-glycans had *m*/*z* ratios consistent with a total of 33 neutral (Hex and HexNAc residues only), 45 fucosylated, 55 sialylated (*i.e.*, Neu5Ac-containing) and 23 sulfated glycans; an additional four glycans possessed both Neu5Ac and sulfate residues (File S1). These results verify that GI MUC2 glycomics can successfully and non-invasively be performed on stool samples in contrast with an earlier report describing 117 *O*-glycans obtained from fetal GI tissues after autopsies (9). No obvious time-dependent trends were apparent in either the numbers of unique glycans in each of these five functionally distinct classes nor in the relative abundance of glycans in each class (Fig. S8). The in terms of glycome composition, most abundant glycans were the sialylated class which comprised an average (standard deviation) of 53% (8.0%) of the total glycan pool, followed by neutral, fucosylated, sulfated, and sialylated and sulfated at 24% (4.5%), 15% (5.0%), 7% (2.8%) and 0.4% (0.22%), respectively. While time-dependent differences for these five groupings of glycans were not apparent, nevertheless, there were persistent, significant differences in these glycan classes apparent between the four individual donors. Summary statistics are provided in Table 1.

**Table 1:** Summary of relative glycan abundances and numbers of distinct glycans in MUC2 collected over 4 months from four individual donors

Class	Donor				*p* (5)
	(A); N = 3	(B); N = 4	(C); N = 4	(D); N = 4	
Neutral					
Abundance (RSD) (1)	20.7 (12.0)^b^	22.7 (20.7)^b^	30.6 (12.0)^a^	22.1 (4.7)^b^	<0.001
# Glycans (RSD) (2)	24.7 (8.4) ^ab^	15.0 (18.1)^c^	27.2 (10.1)^b^	21.2 (11.1)^a^	<0.001
Total # Glycans (3)	32	31	22	28	
Non-detection rate (4)	22.9	12.1	31.8	24.1	
Fucosylated					
Abundance (RSD)	10.6 (23.2)^b^	10.2 (24.0)^b^	18.7 (11.5)^a^	19.4 (12.8)^a^	<0.001
# Glycans (RSD)	23.7 (9.7) ^bc^	14.5 (34.5)^c^	32.3 (16.9)^a^	34.7 (10.9) ^ab^	<0.001
Total # Glycans	32	25	41	40	
Non-detection rate	26.0	42.0	21.3	13.1	
Sialylated					
Abundance (RSD)	61.2 (4.2)^a^	59.9 (7.1)^a^	46.5 (8.1)^b^	47.9 (13.0)^b^	0.001
# Glycans (RSD)	31.7 (6.6)	23.8 (22.3)	27.5 (10.8)	32.3 (20.2)	0.101
Total # Glycans	41	39	38	45	
Non-detection rate	22.7	39.1	27.0	28.3	
Sulfated					
Abundance (RSD)	6.9 (10.4) ^ab^	7.1 (41.6) ^ab^	4.0 (21.2)^b^	10.2 (15.6)^a^	0.005
# Glycans (RSD)	14.0 (18.9)^a^	7.0 (7.3)^b^	16.2 (7.7)^a^	12.8 (18.2)^a^	<0.001
Total # Glycans	18	14	20	19	
Non-detection rate	22.2	48.2	18.75	17.1	
Sial. + sulfated					
Abundance (RSD)	0.6 (17.1)	n.a.	0.2 (79.4)	0.4 (62.9)	0.058
# Glycans (RSD)	0.8 (66.7)	n.a.	1.5 (86.1)	2.5 (40.0)	0.186
Total # Glycans	1	0	3	3	
Non-detection rate	0.0	n.a.	50.0	16.7	

**Notes:** (**1**) Mean relative (%) abundance of all glycans within each unique class for three (donor A) or four (donors B – D) consecutive monthly time points were determined for each donor. No data were imputed. Relative standard deviation (RSD), expressed as a percentage of the mean, is listed in parentheses (**2**). Not all glycans were consistently identified at each time point for an individual donor. The mean number (and RSD) of unique HPLC- and/or MS-resolvable glycans across all four (or three) time points was determined (**3**). The total number of unique glycans in each class detected at least once over four (or three) months for each donor (**4**). The frequency at which specific glycans within a class were not detected for an individual donor was calculated as 100% multiplied by the number of times a glycan was not detected divided by the expected number. Glycans were not counted as missing if they were not present in any samples from a donor; in this instance it was assumed to be genetically not possible for that individual to produce a specific glycan (**5**). Significant differences between donors in mean relative abundance and mean number of unique glycans were evaluated using a one-way ANOVA followed by a *post hoc* Tukey-Kramer test with significant differences (α = 0.05) among means denoted with different letters.

**Figure 5:**
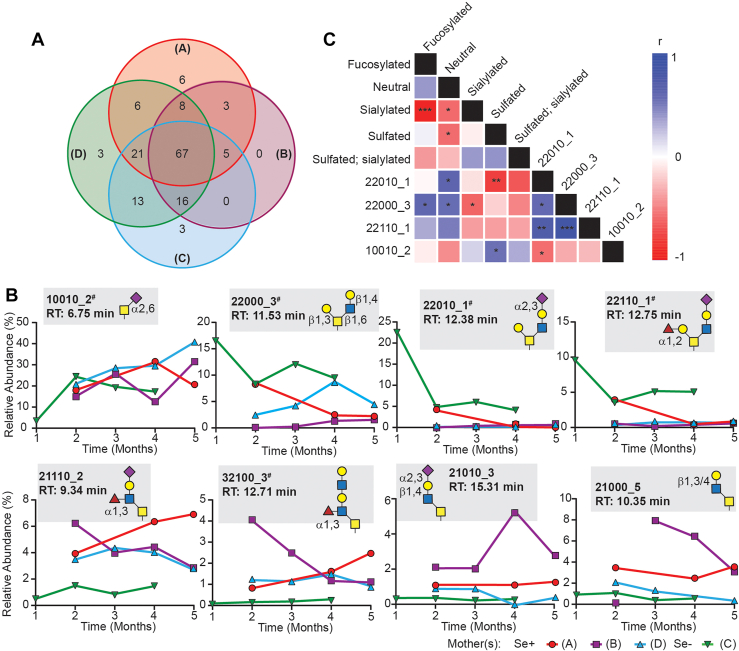
**Neonatal MUC2 glycomics reflected unique, dynamic glycomes.***A*, Venn diagram depicting the numbers of unique glycans detected by HPLC-MS in each of four infants. Qualitatively, very few glycans appeared to be uniquely detected. *B*, semi-quantitative, longitudinal analysis of selected MUC2-borne glycans demonstrates a high variability among four infant GI MUC2 glycomes. Note that each putative *O*-glycan is labelled with a unique six-digit glyco-code with the digits sequentially specifying the number of *m*/*z*-resolvable *N*-acetylhexosamine (HexNAc), hexose (Hex), Fuc, Neu5Ac, and sulfate residues and the _1, _2 suffixes referring to the number of HPLC-resolvable isobars. Glyco-codes followed by the number (#) symbol denotes partial structural annotation by tandem MS (see Fig. S10). Structures are putative but based on established principles of *O*-glycan biosynthesis as well as the previously reported fetal MUC2 glycome. *C*, Spearman’s correlation analysis reveals associations between glycan classes and four glycans accounting for the majority of the variance across all samples (N = 15). Positive correlations are depicted in blue while negative correlations are in *red*; magnitude is indicated by color intensity and significant correlations are denoted ∗, ∗∗, or ∗∗∗ if *p* < 0.05, 0.01, or 0.05, respectively.

Numerically, 99, 116, 126, and 134 glycans were detected in donor B, A, C, and D, respectively. There was considerable overlap between each of the four glycomes in terms of composition, with only a small fraction of the glycans being unique to a single individual (Fig. 5*A*). The large number of glycans shared between individuals suggests that high rates of non-detection for individual glycans for some donors (*e.g.*, B) were due to glycans being below the method detection limit rather than genetic differences preventing the biosynthesis of specific glycans (*e.g.*, unique blood group antigens). While the number of unique glycans and their relative abundances in each of the five compositional classes in each infant remained stable (Fig. S8), there were significant differences in these metrics observed between the four sets of glycomes (Table 1) and when the relative abundances of individual glycans were considered, clear longitudinal differences were observed (Fig. 5*B*). A partial least squares-discriminant analysis (PLS-DA), with samples grouped by individual, was performed in order to identify glycans that discriminated between the four groups (Fig. S9). PLS-DA analysis revealed that four glycans (all included in Figs. 5*B*), 10010_2, 22010_1, 22000_3, and 22110_1, most influenced the separation between the four individuals. Glycan 10010_2, which was putatively assigned as the sialyl-Tn antigen based on its monosaccharide composition and product ion spectrum (Fig. S10), most heavily influenced group separation along component 1 which accounted for the majority of the variation (49.9%) among the samples albeit failing to separate the four individuals. In contrast, component 2, which accounted for only 7.9% of the variance, clearly differentiated between infant A and D, and nearly resolved the four groups of individuals into two unique glycomes, distinguishing between A/D and B/C. Given the competitive nature of *O*-glycan biosynthesis or metabolism, we reasoned that a Spearman’s correlation analysis would more clearly reveal associations between MUC2-derived glycan classes and selected individual glycans. The Spearman-correlation heat map for the five glycan classes and the four most discriminatory glycans identified by PLS-DA is depicted in Figure 5*C*. The Spearman analysis revealed that while 10010_2 accounted for most of the variation among the four individual glycomes, it was only weakly correlated with other glycans and glycan classes and, surprisingly given that 10010_2 contained a Neu5Ac residue, relative abundances were not correlated with the abundance of the sialylated class as a whole even though in some samples 10010_2 accounted for almost 40% of the total detected glycan pool. In contrast, 22010_1, 22000_3, and 22110_1 were all significantly positively associated with each other and the neutral of fucosylated glycan classes while inverse associations were observed between these three glycans and sialylated/sulfated classes. Among entire glycan classes, a clear, significant inverse association was observed between fucose- and Neu5Ac-containing glycans. Consideration of correlations among an expanded list of the 22 most abundant MUC2-derived glycans (Fig. S11) revealed three distinct groups of glycans. Specifically, a group of 22010_1, 22000_3, 22110_1 (all identified by PLS-DA), 21100_4 and 21000_4 all significantly positively associate with each other and fucosylated or neutral glycans while negatively associating with sialylated glycans and a class composed of 20000_2, 31110_2, 11010_3, 31000_2, 21010_4, 32100_3, 21110_2, 21010_3, and 21000_5; putative structures, consistent with those previously reported on fetal GI MUC2 (9), are depicted in Figure 5*B* for 32100_3, 21110_2, 21010_3, and 21000_5. The other abundant glycans considered exhibit less extensive correlations among relative abundances.

### Associations between MUC2 glycosylation in ingested HMOs in breast-fed infants

**Figure 6:**
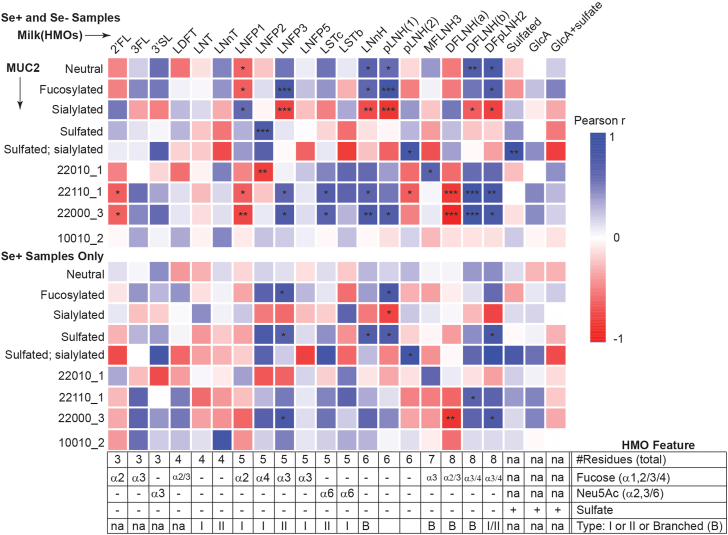
**Correlations between relative abundances of HMOs and the infant MUC2 *O*-glycome.** Spearman’s correlation analysis between relative abundances of selected HMOs or classes of HMOs and infant GI stool-MUC2-borne glycans. Milk and stool samples were collected on the same day. Positive correlations are depicted in *blue* while negative correlations are in *red*; magnitude is indicated by color intensity and significant correlations are denoted ∗, ∗∗, or ∗∗∗ if *p* < 0.05, 0.01, or 0.05, respectively. Data are shown for all samples (N = 15) or infants of Se+ mothers only (N = 11). Only MUC2 glycan classes or individual glycans identified by PLS-DA are depicted; an expanded analysis is presented in Fig. S11. HMOs are organized based on the number of monosaccharide residues and other structural features.

Since HMOs chemically reflect MUC2-borne glycans (Fig. S1*C*), and since our evidence suggested enrichment of unique, sulfate-containing HMOs in the infant gut (Figs. 3*B* and 4*B*), we sought to deduce associations between the relative abundance of sulfated-HMOs in milk (as determined by HPLC-MS) and the abundance of chemically-similar sulfated glycans in infant GI MUC2 (Figs. 6 and S12). Upon considering the entire class of sulfated glycans among both MUC2 and HMOs, a Spearman correlation analysis does not support the hypothesis that high concentrations of sulfated HMOs positively associate with high levels among MUC2. However, these sulfated HMOs are significantly (*p* < 0.01; N = 15) positively associated with MUC2 glycans bearing both Neu5Ac and sulfate residues, a trend also maintained when analyzing only infants fed Se+ milk (N = 11). Significant negative associations were revealed between sialylated MUC2 glycans and LNFP3, LNnH, pLNH, DFLNH(b) and DFpLNH2, conversely, the same HMOs exhibited significant positive associations with fucosylated and neutral MUC2 glycans. LNFP1 meanwhile was positively associated with MUC2 sialylation and negatively associated with neutral or fucosylated glycans. These reciprocal trends for LNFP1, LNFP3, LNnH, pLNH, DFLNH(b) and DFpLNH2 held for glycans 22010_1, 22110_1, and 22000_3, but not 10,010_2, all unique glycans identified by PLS-DA.

## Discussion

The soluble HMOs present in human milk are among the most abundant and diverse across the mammalian species analyzed to date, with absolute concentrations ranging from 10 to 15 g/L throughout the course of lactation and a population in excess of 200 unique structures. Here, we provide HRMS evidence for an additional 24 HMOs that possess *m/z* ratios consistent with monosaccharide compositions that have not been previously described. Nearly all of these novel HMOs bear monosaccharides modified with sulfate residues (Fig. 2 and Table S2). HRMS product ion spectra obtained for seven of these HMOs (Figs. 4*A*, S4. and S5) afford partial structural elucidation. These data extend our earlier report of 15 sulfate-containing HMOs in mature human milk (22). Indirect, albeit more quantitative (than HRMS), evidence in the form of exceptionally high-mobility CE-LIF peaks (Figs 1*B* and 3*B*), further supports the existence of an extended class of sulfated HMOs. We have also previously observed MO sulfation in cow milk (35, 40), while others have reported similar analogues in the milk of dog (50), rat (51), mouse (51), sheep (36), alpaca (36), whale (36), dolphin (36), impala (36), monkey (36, 52), rhinoceros (36), and hippopotamus (36) indicating that MO sulfation may be a feature shared by all mammals. Our investigation of sulfated MOs in cow milk indicated that their relative abundance increased during early lactation (40), rising to a median (*N* = 20) of 36% of the total MOs distinguished by CE-LIF by 1-week *postpartum* (35). In the present study, a slight increase in relative concentrations of all sulfated HMOs (*p* = 0.181; one-way ANOVA) and 3′SO_3_L (*p* = 0.059) in Se + donors sampled at 2, 3, and 4 months *postpartum* (Fig. 3*B*) is suggestive that a similar enrichment to that observed in dairy cattle occurs in humans, although the unavailability earlier sampling time-points prevents a direct comparison to the cow data.

More recently, we, and others, have also reported the mass spectrometric detection of novel uronic acid-containing MOs in cow (35) and a range of other non-domestic mammals (36). We presume that the uronic acid residue present in these MOs is D-glucuronic acid (GlcA) as other common monosaccharides of this class, D-galacturonic (GalA), D-mannuronic (ManA) and L-iduronic acid (IdoA), are usually found in large polysaccharides occurring in plants (GalA) or algae (ManA), or glycosaminoglycans (GAGs) like heparan (IdoA). GAGs also contain abundant GlcA residues, as does the large laminin-binding domain of α-dystroglycan (53). Here, we extend these observations to humans, providing the first HRMS evidence for 22 putative GlcA-containing HMOs in the milk of both Se+ and Se- individuals. Due to the limited availability of samples, the HRMS data for GlcA-containing HMOs was derived from two separate sample sets that were unable to be directly compared: a cross sectional sample set (12 Se+ and 4 Se-) collected at 5 months *postpartum* (Fig. 2, *B* and *C*) and a longitudinal set (3 Se+ and 1 Se-) of milk samples collected at monthly intervals (Table 2, Fig. 4*A* and Table S2). As in both aforementioned studies (35, 36), glucuronyl-β1,4-lactose (GlcA-L; glyco-code = 020010), a glycan marketed as a precursor for the synthesis of the human natural killer cell-1 (HNK-1) antigen (also known as CD57) and not specifically as an HMO standard, was detected in most samples, however, solely GlcA-bearing HMOs were less abundant in terms of both numbers of unique structures and relative concentrations than HMOs possessing both sulfate and GlcA residues (Fig. 2, *B* and *C*). Among the cross-sectional data set, GlcA-L does not appear to be the biosynthetic precursor of the majority of GlcA-bearing HMOs since a larger sulfate- and GlcA-containing trisaccharide (030011) and three putative derivatives thereof (030111_1, 030111_2 and 031111) were more frequently detected and found at higher relative abundance than GlcA-L and its putative derivatives (020011_1, 020011_2 and 021010; Fig. 2, *B* and *C*). Three 030011 isobars were detected among the longitudinal data set although only one (030011_1) was detected in all 15 samples (Table S2), and we thus hypothesize that the 030011_1 detected in the longitudinal data set is equivalent to the single 030011 isobar identified in the cross-sectional data set. Given that among the cross-sectional samples 030011 relative abundance was significantly higher in Se-milk, and that median levels of a related 031111 were also higher in Se- samples (*p* = 0.058, Mann–Whitney *U* test), we hypothesize that both glycans shared a common β3′ or 6′-galactosyllactose core modified by a sulfated GlcA residue as depicted for HMOs 031,111_4 and 030111_3, two HMOs for which product ion spectra have been obtained (Fig. 4*A*). In support of this assignment, we note that absolute concentrations of β3′or 6′-galactosyllactose (glyco-code = 030000) were both higher in Se- milk than Se+, with both HMOs remaining stable over a 12-month lactation period (54). It should also be noted that the GlcA-transferase responsible for the biosynthesis of CD57—classically defined as the 3-*O*-SO_3_-GlcA-β1,3-Gal-β1,4-GlcNAc-R trisaccharide where R is an *N*- or *O*-glycan (55) or glycolipid (56)—has been shown to use both β1,3- and β1,4-linked Gal residues as acceptor substrates (57), suggesting a somewhat relaxed substrate scope. Meanwhile, the biosynthesis of all GAG classes (with the exception of hyaluronic acid) is initiated *via* a protein-linked tetrasaccharide primer consisting of GlcA-β1,3-Gal-β1,3-Gal-β1,4-xylose (Xyl) (58) in which the reducing end moiety (Gal-β1,4-Xyl) structurally mimics lactose (Gal-β1,4-Glc) with the exception that the Xyl lacks the 6-hydroxy-methylene (CH_2_OH) group of the Glc residue. Xylosides have been shown to prime protein-free GAG biosynthesis (59) and we thus hypothesize that the high concentration of lactose in the mammary gland may similarly deflect the activity of a GAG-specific GlcA-transferase to form GlcA-β1,3-Gal-β1,3-Gal-β1,4-Glc (*i.e*. 030010), from which a series of sulfated HMOs are then produced. Note that GAG linkers modified with sulfate, fucose, Neu5Ac and/or Neu5Gc residues (60, 61, 62) have previously been reported on glycoproteins like bikunin and brevican, proteoglycans with roles in regulating inflammation and neural plasticity. Accordingly, a range of CE-LIF and tandem MS experiments was used to demonstrate that human GlcA- and Fuc-transferase enzymes are capable of synthesizing CD57-and/or GAG-core-tetrasaccharide-like HMOs (Figs. S15 and S14).

**Table 2:** List of paired, longitudinal milk and stool samples analyzed

Donoronor^(a)^	Sex (baby)	Se + or Se- (mother)^(b)^	Months *postpartum*	
			HMO (milk/stool)^(c)^	MUC2 (stool)
A	Male	Se+	2–5	2–5
B	Male	Se+	2–5	2, 4, 5
C	Female	Se-	2–4	1–4
D	Female	Se+	2–5	2–5

**Notes:** (**a**) All babies were born vaginally. (**b**) Secretor status (Se+ = positive; Se- = negative) was determined by the relative abundance of 2′FL, LDFT, and LNFP1; Se-individuals produce only trace (or less) concentrations of these HMOs. (**c**) CE-LIF analysis of HMOs in breast milk and stool was performed with samples collected at 2 to 4 months; HPLC-MS analysis was used for HMOs samples collected from 2 to 5 months with the exception of donor C.

Where the functions of GlcA and/or sulfate-containing glycans have been described, they typically involve cell-cell interactions especially in the immune system. For example, GlcA-containing MOs have recently been shown to attenuate cytokine production by activated macrophages (36), while both sulfated MOs (63) and GlcA-containing lipids (56) are able to block the leukocyte-selectin interactions necessary for their homing to sites of inflammation. However, any putative immunomodulatory functions of these sulfate/GlcA-containing HMOs depend on their resistance towards microbial metabolism, which would substantially diminish their effective concentrations. This necessary condition leads to two linked hypotheses. First, since both GI MUC2- (34) and proteoglycan-borne (64) glycans require de-*O*-sulfation as a key regulatory step for metabolism, it was hypothesized that sulfated HMOs would be enriched in the infant gut relative to non-sulfated analogues. Second, since major GI microbes use identical pathways (16) to access HMOs and MUC2-glycans to meet their metabolic requirements, high concentrations of sulfated HMOs may deflect the microbiome’s metabolism away from MUC2-borne glycans leading to detectable changes in the host glycome. The CE-LIF data presented herein (Figs. 3 and S3) indicate that HMOs with CE mobilities consistent with sulfated HMOs are highly—up to 20-fold in some individuals—enriched in stool relative to neutral or sialylated HMOs in infants fed both Se+ and Se- milk across at least 4 months *postpartum*. The magnitude of this enrichment of sulfated HMOs in the infant GI tract may be underestimated for two reasons: first, the ambiguity in identifying sulfated HMOs larger than trisaccharides due to their co-migration with small, neutral HMOs and, second, due to the co-migration of highly charged HMOs (*i.e.*, those simultaneously bearing sulfate, GlcA, and/or Neu5Ac residues) with excess fluorogenic-labelling reagents. While the CE-LIF (Fig. 3) and HPLC-MS (Figs. 4 and S6) are both consistent in identifying LNT and 2′FL as preferred prebiotic HMOs, mass spectral evidence for the enrichment of sulfated HMOs is more variable, with some samples exhibiting sulfated HMOs as the most abundant in stool while being undetectable in others collected at the same time point. The variability in detecting highly anionic HMOs in negative ion mode mass spectrometry may, in part, be due to the conditions used. Consistent with previous studies, MS was performed after HPLC separation of HMOs using a porous graphitic carbon (PGC) stationary phase operated in reverse-phase conditions with formic acid- (22, 35, 40, 43, 46) or ammonium bicarbonate-containing (36) eluents. We have demonstrated that under the more widely used acidic conditions, highly sulfated glycans are inconsistently detected by MS (65). Notwithstanding the potential for negative bias against sulfated HMOs, the HPLC-MS data indicate that sulfate-containing HMOs are enriched in stool (especially at 4 and 5 months *postpartum*), consistent with the more quantitative CE-LIF data, thus supporting the first hypothesis that sulfation confers metabolic resistance to HMOs leading to an increase in relative abundance in the infant GI tract.

While the variation of HMOs in milk and their recovery from stool samples has been previously reported (13, 41, 42, 43), only a few of these have analyzed HMOs from matched mother-infant pairs collected on approximately the same day (13, 42). Noting that studies employing MS-based detection are usually limited to a few commercially available HMOs, Albrecht and colleagues employed a less selective technique, CE-LIF, to quantitatively compare concentrations of APTS-labelled HMOs extracted from 11 matched milk and stool samples collected monthly over 6 months (42), achieving partial HMO structural annotations *via* pairing CE-MS with in-line LIF detection (41). While the sampling timepoints, extraction and analysis conditions used by Albrecht *et al.* (41, 42) were comparable to those reported herein, these authors did not note the enrichment of highly mobile, putatively sulfate/GlcA-containing HMOs observed in our study. However, there are several non-exclusive reasons that may account for these divergent results. First, Albrecht *et al.* used polymer-coated capillaries, whereas those used here were fused silica and thus capable of generating a slight electroosmotic flow absent under the Albrecht *et al.* conditions. Second, although beginning with the same proprietary background electrolyte, Albrecht *et al.* manually reduced the pH (by roughly two units) to facilitate comparison between CE-MS and CE-LIF, an adjustment that may have affected migration times especially of acidic glycans; for example, we note that the two CE methods reversed the migration times for lactose and 3′SL while the neutral pair of 3FL and 2′FL co-migrated under both sets of electrophoretic conditions. Third, while both reports employed PGC to desalt HMOs and remove most neutral mono- or disaccharides prior to APTS-labelling, we note that our conditions doubled the trifluoroacetic acid (TFA) concentration of the eluting solvent, possibly accounting for increased yields of the highly anionic glycans, which we have previously demonstrated require highly acidic eluant additives for optimal recovery (66). Finally, we note that careful inspection of the Albrecht *et al.* CE-LIF data collected from stool-extractible HMOs (42) indicate numerous, unannotated peaks migrating ahead of their ISTD (xylose) in addition to vaguely defined peaks labelled “monomers”; interestingly, as observed here (Fig. 3), these highly mobile CE-LIF peaks accounted for a substantial fraction of the total HMO pool in stool while they were barely detectable in milk samples. Thus, we conclude that these prior, comparable studies provide previously unrecognized CE-LIF evidence for the existence of highly anionic HMOs that uniquely survive in the infant GI tract.

Independent of the enrichment of sulfated HMOs in the infant GI tract, the CE data reported by Albrecht *et al.* (41, 42) indicated the presence of glycans in stool that contained glyco-epitopes like blood group antigens that were not apparent in the ingested milk. HPLC-MS analyses (comparable to those reported herein) by Davis and colleagues (43) have likewise identified free *N*- and *O*-linked glycans in the stool of infants, attributing their presence to the activity of microbial endo-glycosidases. While our CE-LIF data likewise revealed the presence of low mobility (*i.e.* large, uncharged) glycans, characterizing these was beyond the scope of the present study which instead focused on assessing whether, first, MUC2 could be purified from infant stool samples after homogenization and second, whether the soluble milk-derived HMOs could deflect the microbiome’s glycolytic activity away from MUC2-bound glycans. Frese *et al.* have provided indirect evidence in support of this hypothesis in a probiotic study in which *B. infantis*-fed infants exhibited much higher rates of HMO consumption than the non-inoculated control group (46) with correspondingly lower concentrations and diversities of free, putatively mucus-derived *O*-glycans in stool (47). However, as milk glycoproteins contain abundant *N*- and *O*-linked glycans, it is uncertain whether these free glycans were diet- or host-MUC2-derived. To eliminate this source of ambiguity, stool-adherent MUC2 was highly enriched (Fig. S7) on the basis of its differential-solubility in guanidinium hydrochloride before and after disulfide reduction (10, 37) prior to glycomic analysis (Fig. 5, Table 1, Figs. S8 and S10, and File S1). Our experimental approach substantially advances earlier glycomic research on the neonatal/infant GI tract which has so far focused on the analyses of glycoproteins isolated from either human meconium (3, 4, 5, 6) or from fetal GI tissues (9). Specifically, the research herein clarifies and advances these previous studies in four ways as it is the first to unambiguously analyze GI MUC2 (*vs.* undefined glycoproteins), report semi-quantitative data for all glycans, perform HRMS after HPLC separation of isobaric glycans (*vs.* MALDI MS, which fails to resolve isobars, or off-line MS analysis in which retention times were uncoupled from qualitative data), and afford longitudinal analysis (enabled by the non-invasive sample collection). Capon *et al.*, obtained pools of *O*-glycans after performing reductive β-elimination on water-soluble meconium-derived glycoproteins (5); although the physio-chemical properties (*i.e.*, water-solubility) of these glycoproteins differs from gel-forming MUC2—which is unsurprising since animal models have clearly indicated that post-natal GI colonization is required to induce the secretion of guanidium-insoluble MUC2 (67)—it is probable that the material analyzed by Capon *et al.*, is unpolymerized MUC2 given its ubiquitous expression throughout the fetal GI tract (68). Detailed qualitative analyses of 10 of the 80 unique putative *O*-glycans liberated from meconium glycoproteins by Capon *et al.* revealed that most were based on core-1 structures (Fig. S1*B*) with Neu5Ac predominantly α2,6-linked to the reducing end GalNAc (6). Comparable and concurrent research by Hounsell *et al.* revealed the presence of core-5 and 6 *O*-glycans on meconium-derived glycoproteins (3, 4), rarer core structures that still await systematic structural and functional investigation; note that these studies (3, 4) were restricted to de-sialylated glycans. More recently, Robbe-Masselot *et al.*, have obtained MS evidence for putative core-5 *O*-glycans among the predominantly core-2 structures derived from the water-soluble mucus collected from different regions of the fetal GI tract (9). For example, using HPLC-MS and MS/MS, Robbe-Masselot identified two glycans, each with two isobars, with *m/z* consistent with glyco-codes 20000 and 20010. The former, 20000 (containing two HexNAc residues), could plausibly be either a core-1, 5, or six glycan (given the earlier findings of Hounsell *et al.*) although discriminating between these possibilities was not possible based on MS/MS alone; however, 20010 (with two HeNAc and one internal Neu5Ac residue) can only plausibly be considered a core-5 glycan based on our current understanding of *O*-glycan biosynthesis. Among the infant stool samples analyzed in present study, two 20000 isobars were detected, both being included among the most abundant 25 glycans in the data set (File S1); we have likewise identified multiple 20000 isobars in comparable adult MUC2 samples (37), but in neither instance were 20010 isobars detected. However, our MUC2 results are broadly consistent with these previous studies in that most structures, where these could be partially elucidated by MS/MS (Fig. 5*B* and Fig. S10), were core-1 or core-2 glycans.

As noted, ours is among the first human GI MUC2 glycomic data set—either adult- or fetal/infant-derived—that includes semi-quantitative data for all glycans across biological replicates. For example, Larsson *et al.*, (8) reported the glycome of guanidinium-insoluble MUC2 (obtained from adult colonoscopies), providing semi-quantitative data for 17 unique *m/z*—albeit with all isobars unresolved with the exception of the above noted 20010 pair—for 25 patients. Although brief in (semi)quantitative details, Larsson *et al.*, did, however, perform in-depth qualitative analyses that demonstrated that the majority glycans, in terms of unique structures (not quantities), derived from the adult colon MUC2 bore core-3 glycans that are thus distinct from fetal/infant MUC2. Therefore, the data reported herein provide a benchmark for both the *O*-glycan diversity, relative abundances, and inter-individual variation for infant-derived MUC2 while the sample preparation methods (10, 37) and non-invasive nature of sample collection will afford researchers a reasonably-accessible method to address when and how the shift from a neonatal to an adult GI MUC2 glycome occurs as well as enabling investigations of the functional consequences of these phenomena. Studies semi-quantitatively comparing infant and adult colon/stool-derived MUC2 glycans are currently in progress. The intra-individual longitudinal stability in infant MUC2 in terms of both diversity and relative abundance of broad glycan classes defined on the basis of unique monosaccharide constituents (Fig. S8) indicates that at least over the first 5 months *postpartum*, neither biosynthetic nor metabolic (*i.e*., microbial) factors influence the fucosylation, sulfation, or sialylation of *O*-glycan core-structures and that each infant’s glycome exhibits stable inter-individual differences (Table 1); these core-modifications are thus likely genetically influenced. This high-level stability within the infant MUC2 glycome may also account for our inability to deduce the hypothesized associations between relative HMOs abundances and MUC2 glycan classes beyond the positive association between sulfated HMOs and sulfated and sialylated MUC2 glycans (Fig. 6). For example, the abundant fucose-containing HMO 2′FL was not correlated with fucosylated MUC2 glycans while equally abundant LNFP1 (Fig. S3), that, like 2′FL bears a single α1,2-linked Fuc, was significantly inversely associated with MUC2 glycosylation but positively associated with sialylation. Since both 2′FL and LNFP1 are major probiotics, exhibiting large reductions in abundance in nearly all stool samples (Figs. 3*B* and S3), the lack of (2′FL), or inverse (LNFP1), correlations with MUC2-borne fucose suggests that these HMOs neither provide MUC2-secreting goblet cells with a dietary Fuc source nor detectibly deflected microbial fucosidase activity from secreted MUC2 glycans. Furthermore, consistent and significant associations between individual glycans and structurally-unrelated HMOs (Figs. 6 and S12) indicates that these correlations are likely reflecting the high degree of co-variation in MUC2 glycans due to the competitive nature of glycan biosynthesis which, given the noted stability in glycan classes as defined by terminal monosaccharide residues, is hypothesized to be due to competition between the glycosyltransferases responsible for generating the different *O*-glycan cores. In support of this, we note that three of four abundant glycans (Fig. 5*B*) identified by PLS-DA as most discriminating between individuals (22000_3, 22010_1 and 22110_1), all of which were verified by tandem MS as core-2 *O*-glycans (Fig. S10), significantly positively correlated with each other (Fig. 5*C*) while negatively correlated with a group of at least nine glycans (Fig. S11) that included 21110_2, 32100_3, 21010_3 and 21000_5. The relative concentrations of these nine glycans almost all significantly positively correlated with each other as well as sulfated and sulfated/sialylated glycans more broadly and are thus hypothesized to be core-3 *O*-glycans as depicted for 21110_2, 32100_3, 21010_3 and 2100_5 in Figure 5*B*, an assignment that could be verified for 32100_3 by tandem MS. Although verification of these classifications awaits the completion of ongoing structural studies, they are consistent with the principles of *O*-glycan biosynthesis in which sialylation of the reducing end GalNAc of *O*-glycans is possible for core-3 but not core-2 structures, thus accounting for the clear reciprocal associations between these classes and total sialylation. Meanwhile, sialylation of the reducing end GalNAc prior to core formation (forming sTn, *i.e.*, 10010_2) is a biosynthetic dead-end blocking all further glycan elongation which may explain why 10010_2, the most abundant glycan in most samples, was correlated with very few other glycans within the data set (Fig. S1*B*).

In summary, the research reported here extends previous glycomic studies on infant (3, 4, 5, 6, 9) and adult (8) colon/stool-derived MUC2 glycosylation, especially in affording important semi-quantitative details. The glycomic data currently do not indicate that an obvious switch from infant-like core-1/2 glycosylation to adult-like core-3/4 structures occurs before five months *postpartum* (at least in exclusively breast-fed infants). Verifying this conclusion requires the completion of ongoing qualitative studies as well as investigation of a larger cross sectional population, both activities that will be enabled based on our successful demonstration that glycomics can be achieved with non-invasively-collected stool samples previously processed (*i.e.*, homogenized) for other tests such as meta-genomics, immuno-assays for cytokines (22, 39), or fecal calprotectin quantitation. Some GI microbes that persist in the adult gut preferentially adhere to core-3 glycans (69) and thus the glycomic changes occurring during infancy are undoubtedly relevant to the acquisition of a stable, adult-like GI microbiome. In this respect, it should be noted that at least some microbial endo-*O*-glycanases (48) and glycoproteases (70) are likewise glycan-dependent. Our MUC2 extraction protocol permitted the simultaneous extraction and subsequent analysis of HMOs that had survived microbial metabolism or absorption and the data presented herein are consistent with previous studies analyzing paired milk/stool samples (13, 42). While associations between ingested HMOs and the infant GI MUC2 glycome were not observed, our data demonstrate that highly anionic HMOs, with CE mobilities consistent with sulfated structures, are significantly enriched in the stool in accordance with the hypothesis that sulfation generally confers metabolic-resistance to glycans against microbial glycosylhydrolases. HRMS and MS/MS data for sulfated HMOs are reported, extending a previously observed class (22) collected from both Se+ and Se- individuals and we report, for the first time, evidence for GlcA-containing HMOs in humans, many of which are also sulfated. Aside from high molecular weight GAGs, GlcA-containing glycans have been considered rare in nature; we propose that the monosaccharide composition (deduced by HRMS) and partial regiochemical assignments (afforded by MS/MS) are consistent with the GAG linker tetrasaccharide that has been primed by lactose rather than the usual Ser/Thr-linked xylose. The enzymatic synthesis of several of these novel structures from known HMO precursors further supports the lactose-priming hypothesis. We expect to uncover intriguing bio-activities for this new class of HMO given the immune-modulating properties of analogues isolated from other mammals (36) and given their persistence through the infant GI tract.

## Experimental procedures

### Stool and breast milk collection

All samples were collected according to the Declaration of Helsinki guidelines, and all procedures were approved by the University of British Columbia Clinical Research Ethics Board and the British Columbia Interior Health Ethics Board. Informed, signed consent was obtained from each participant at enrollment. All samples were initially collected in the Okanagan Valley, Canada, as part of a prospective clinical study; the full study design and initial findings have been previously published (71). Prior to enrollment, participants were screened to only include exclusively breastfeeding mother-infant pairs; infant diagnosis with a disease or premature birth were exclusion criteria. Infant stool collection, storage, and processing details have been described in detail elsewhere (72). Likewise, breast milk collection protocols have been previously described (22). Both milk and stool samples were collected on the same day, and both were stored in the participants’ home freezer (*ca.*, −20 °C) for up to a maximum of 3 days, after which samples were transported to the research facility on dry ice to be stored at −80 °C until further analysis. Two sets of samples were analyzed herein: first, a cross-sectional set of *N* = 16 (12 Se+ and 4 Se-) breast milk was used for HMO analysis by HPLC-MS and CE-LIF as described previously (22); all of these samples were collected at 5 months *postpartum*. A second, longitudinal set of samples was used for HMO analyses in both milk and stool as well as for MUC2 glycomics; this longitudinal sample set included both infant stool and breast milk collected from four infant–mother pairs. The paired milk/stool samples that were available for analysis are recorded in Table 2.

### Chemicals, reagents, and general experimental details

Unless explicitly noted, all chemicals were purchased from MilliporeSigma and used as received. The following chemicals were of analytical grade or better: HPLC-MS grade methanol (MeOH), HPLC-MS grade acetonitrile (ACN), trifluoroacetic acid (TFA), acetic acid (AcOH), formic acid, ammonium bicarbonate (NH_4_CHO_3_) and sodium cyanoborohydride (NaBH_3_CN). cOmplete protease inhibitor tablets (10×) were also purchased from MilliporeSigma. 18 MΩ cm water was provided by a Barnstead E-Pure water purification system and was used for all preparative analytical procedures unless otherwise specifically stated. 8-aminopyrene-1,3,6-trisulfonate (APTS) was synthesized in-house and purified exactly as previously described (66) and stored dry until reconstitution to a final concentration of 100 mM in 0.9 M citric acid; working stocks of APTS were stored at −20 °C. All HMO standards were purchased from Carbosynth (now Biosynth) with the exception of GlcA-L (Elicityl) and maltoheptaose (Toronto Research Chemicals); all standards were used as received. Note that LSTc was determined to be a mixture of isobars, LSTc and LSTb as deduced by tandem MS (Fig. S2); similarly, pLNH contained a mixture of two isobars, although these were not further assigned. A mixture of Glc-containing oligomers, spanning a monomer (*i.e.*, 1 glucose unit; GU) to at least 20 GUs, was synthesized to afford a CE-LIF glycan-mobility standard. Briefly, corn starch (purchased from a local grocery store) was dissolved in 0.1 M HCl to 49 mg/ml and hydrolyzed for 15 min at 100 °C upon which it was snap frozen and immediately dried *in vacuo*. Supelclean ENVICarb porous graphitic carbon (PGC) solid-phase extraction (SPE) cartridges (250 mg; 3 ml bed volume) were purchased from MilliporeSigma; these were always conditioned by washing with 80% ACN containing 0.1% TFA (3 ml) followed by water (6 ml) prior to use. Samples were routinely dried *in vacuo* using a Savant SPD121P SpeedVac centrifugal concentrator connected to a Savant RVT5105 refrigerated vapor trap (ThermoFisher Scientific) and/or lyophilized using a Labconco Freezone 4.5 freeze-dry system. Dialysis procedures were performed using a 2 L Spectraflo Dynamic Dialysis System (Repligen) connected to a 50 L dialysis reservoir. Dialysis tubing (100 kDa nominal molecular weight cut-off; NMWCO; 22 mm diameter) was primed in 20% isopropanol for 5 to 10 min before use. Polyvinylilidene difluoride (PVDF) membranes, Pierce protein-free Tris-buffered saline with Tween-20 (TBST) blocking buffer, horseradish peroxidase (HRP)-conjugated streptavidin, HRP-conjugated donkey anti-rabbit IgG, and Pierce SuperSignal Enhanced Chemiluminescent reagent were all purchased from Thermo Scientific. The protease inhibitors leupeptin, E−64 and 4-(2-aminoethyl)benzenesulfonyl fluoride hydrochloride (ABESF) were all purchased from Bioshop Canada. The following biotinylated lectins were acquired from Vector Laboratories: *Ulex europeaus* agglutinin 1 (UEA1), *Maakia amurensis* lectin 2 (MAL2), *Sambucus nigra* agglutinin (SNA) and Jacalin (JAC). Finally, the following recombinant human glycosyltransferases were obtained from R&D Systems: β1,3-glucuronyltransferase 1 (BGAT1), β1,3-glucuronyltransferase 3 (BGAT3), fucosyltransferase 3 (FUT3), and fucosyltransferase 2 (FUT2). The following nucleotide sugars were purchased from MilliporeSigma: uridine-5′-diphosphate (UDP)-GlcA, and guanosine-5′-diphosphate (GDP)-Fuc.

### HMO extractions

HMOs were extracted from 1 ml of all new milk samples (*i.e.*, the longitudinal set) using a combination of liquid-liquid and solid-phase extraction (SPE) exactly as previously described (22, 40). Each sample was fortified with 20 μg of the maltoheptaose ISTD after protein and lipid removal by LLE but before desalting and lactose reduction by ENVICarb SPE. Partially enriched, ISTD-fortified HMO samples were loaded onto conditioned ENVICarb cartridges in 500 μl H_2_O after which each cartridge was sequentially washed with H_2_O (10 ml), 5% ACN (6 ml), 20% ACN (6 ml) and 50% ACN containing 0.1%TFA (6 ml). All SPE procedures were performed using positive pressure. The 20% ACN and 50% ACN + TFA fractions were pooled, partially concentrated using a SpeedVac, and lyophilized. To extract non-metabolized HMOs from stool samples, the samples, which had been homogenized in preparation for metagenomic analysis (72), were first lyophilized to account for variable amounts of water; 10 mg of this dried material was mixed with 100 μl H_2_O at 4 °C overnight and any insoluble materials were removed by centrifugation (4000*g*, 5 min). The water-soluble material was extracted exactly as the milk samples described above, except scaled down in volume, that is, lipid removed by Folch-extraction with four volumes of 2:1 chloroform:methanol followed by a cold ethanolic protein-precipitation of the remaining aqueous phase, which was subsequently dried and desalted by ENVICarb SPE. Stool-derived samples were, like the milk, fortified with 2 μg ISTD immediately before SPE. Separate HMO extractions were performed for the CE-LIF and HPLC-HRMS sample sets (Table 2). Purified HMO samples were stored at −20 °C prior to further treatment or analysis.

### MUC2 extraction and glycan reductive β-elimination

Mucus was extracted using a method previously published with minor changes (37). Freeze-dried fecal material was resuspended in 5 ml mucus extraction buffer (MEB: composed of 6 M guanidinium hydrochloride (GuCl), 0.1 M Tris, and 1 mM EDTA, pH 8.0) containing an in-house protease inhibitor cocktail composed of 5 μM E64, 10 μM leupeptin, and 250 μM AEBSF. Depending on availability, fecal material between 9 and 110 mg was taken for the extraction. After adding the MEB, samples were placed on an orbital shaker set to maximum rotation overnight at 4 °C after which insoluble material was pelleted by centrifugation at 3500*g*, 4 °C for 20 min. The supernatant was carefully removed post-centrifugation and discarded. The gel-forming MUC2-containing pellet was resuspended in an additional 5 ml of MEB-containing protease inhibitors and placed back on the rotator for 4 hours after which the centrifugation and supernatant removal steps were repeated. After a third round MEB-assisted removal of GuCl-soluble material, the disulfide bonds in the remaining pellet were reduced by the addition of freshly prepared dithiothreitol (DTT) in MEB to a final concentration of 100 mM. Samples were agitated at 37 °C for 16 h after which another aliquot of DTT was added to bring the concentration to 200 mM. After reduction for an additional 6 h, samples were alkylated by adding iodoacetamide to a final concentration of 300 mM; alkylation was done for 16 h at room temperature and protected from light using aluminum foil. Insoluble material remaining after alkylation and reduction was removed by sequentially passing samples through nylon syringe filters (Thermo Scientific) of 125 and 5 μm pore size. Low molecular weight materials were then removed from the reduced, alkylated, and GuCl-solubilized MUC2 by dialysis against a 100 kDa molecular weight cut-off (MWCO) membrane against reverse osmosis purified water that was continuously refreshed at a flow rate of ∼300 ml/min. Dialysis was done overnight, after which samples were transferred to 15 ml tubes and freeze-dried.

With the exception of two samples (from donor B and D, both at 4 months *postpartum*) in which a small portion of the extracted MUC2 was reserved for immuno- or lectin-blot analysis, the entirety of each purified MUC2 sample was subjected to reductive β-elimination following Carlson’s classic procedure (10, 37, 49). In brief, samples in water were transferred to 1.5 ml centrifuge tubes, lyophilized and dissolved in a solution of freshly prepared 50 mM NaOH containing 1 M NaBH_4_ and heated at 45 °C for 16 h. After cooling, the reactions were quenched by carefully adding glacial acetic acid dropwise until all fizzing stopped. The quenched solutions were directly applied to conditioned ENVICarb SPE cartridges that were sequentially washed with 5 ml H_2_O and eluted with four serial washes (600 μl each) of 50% ACN containing 0.1%TFA; these washes, containing the desalted, reductively-eliminated MUC2 *O*-glycans, were pooled, partially concentrated using a SpeedVac concentrator, lyophilized, and stored at −20 °C until HPLC-MS analysis.

Composite sodium dodecyl sulfate (SDS)-urea-agarose polyacrylamide-gel electrophoresis (UAgPAGE; (37)) was used to resolve MUC2 collected from two samples to verify that the adapted extraction protocols suitably recovered high molecular weight MUC2 from homogenized stool samples. Porcine stomach mucin (type 3; MilliporeSigma) was used as a positive control while bovine serum albumin, which is not glycosylated, was used as a negative control. After electrophoretic separation, samples were transferred to PVDF membranes, blocked with protein-free TBST blocking solution and probed with biotinylated UAE1, MAL2, SNA, or JAC (all at 2 μg/ml) in TBST at 4 °C for 16 h; after washing the blots, bound lectins were detected using streptavidin-linked horseradish peroxidase (1:200 dilution; 1 h incubation at room temperature). Alternatively, rabbit polyclonal anti-MUC2 antibodies (anti-LS174T; (37)) diluted 1:2000 in TBST containing 5% skim milk powder were used for immunoblot analysis of MUC2; bound antibody was detected using HRP-conjugated goat anti-rabbit IgG (1:2000). Bound lectins/antibodies were detected with SuperSignal Enhanced Chemiluminescent reagent following the manufacture’s (Thermo Scientific) instructions and imaged using a ChemiDoc MP system (BioRad).

### CE-LIF analysis

Milk- or stool-derived HMOs intended for CE-LIF analysis were carefully lyophilized in 200 μl micro-tubes and derivatized with APTS exactly as previously described (22, 35, 40). Post-derivatization, samples were diluted to a final volume of 100 μl in H_2_O and analyzed using a ProteomeLab PA800 (Beckman Coulter) operating in reverse polarity (−30 kV) using 50 μm internal diameter fused silica capillaries (44 cm to detector; 50 cm total length) and a proprietary NCHO buffer (SCIEX) as the background electrolyte; this buffer was replaced after approximately every 20 samples. All peaks not attributable to the APTS labelling reagents in the absence of sample were manually integrated using 32 Karat software (Beckman Coulter); peaks migrating after the ISTD to the end of each run were very difficult to align, and this region was integrated in aggregate. Where HMOs were not apparent, especially in the stool-derived samples, a relevant region of the baseline electropherograms was integrated. HMOs were assigned structures based on their co-migration with commercial standards. Peak areas were normalized to the total integrated area, which did not include lactose or several unique, highly mobile peaks eluting among the APTS labelling reagents. Post-integration processing was performed in Microsoft Excel. Although relative abundances of some MOs in the human (22) or bovine (35) cross-sectional data sets have been reported previously, in neither instance was an unambiguously sulfated CE-LIF region clearly defined due to the earlier lack of the disialyllactose (DSL; bovine) and GlcA-L and DSL (human) standards. Representative electropherograms were exported as text files using 32 Karat software and subsequently plotted using OriginLab (version 9.7).

### HPLC-MS analyses

#### General HPLC-MS details

HPLC (Agilent) separations were performed with a 1290 Infinity system supplied with a binary pump, autosampler and temperature-controlled column and sample compartments maintained at 40 and 4 °C, respectively. The injector needle was washed with a solution of isopropanol: MeOH: H_2_O (2:1:1) for 3 s after each injection cycle. HRMS was performed using a 6530 quadrupole time-of-flight (QToF) equipped with a Jet Stream electrospray ionization source (Agilent). All mass spectral data were acquired in negative ion mode with the following source parameters: drying gas (N_2_) temperature 300 °C with a flow rate of 10 L/min; sheath gas (N_2_) temperature 400 °C with flow rate of 12 L/min; nebulizer pressure 45 psig; capillary voltage 4750 V; nozzle voltage 1000 V; and fragmentor voltage 175 V. The QToF was tuned and calibrated in the 2 GHz extended dynamic range mode for the 100 to 3200 *m*/*z* range prior to all sample analyses. Full-scan spectra were collected at a rate of 2 Hz and saved in profile format. Instrumental drift was accounted for by using a reference ion solution (Agilent) containing 10 μM purine (*m/z* 119.0360 for [M-H]^-^) and 2.0 μM HP-0921 (*m*/*z* 966.0007 and 1033.9881 for [M-H]^-^ and [M + HCOO]- ions, respectively) diluted in 95% ACN; this reference ion solution was delivered to the HPLC eluent post-column at 800 μl/min by a 1260 Infinity II isocratic pump (Agilent) with a flow-splitter sending 1/16th of the solution to the MS with the remaining getting recycled. Post-acquisition analysis was performed using MassHunter Workstation software (Agilent), including Data Acquisition Workstation (v B.06.01, SP1) and Qualitative Analysis (v B.07.00, SP2). The Find-by-Formula algorithm in the Qualitative Analysis software was used to survey the HRMS data for formula matches within a ± 10.00 ppm mass accuracy limit and, when applicable, a ± 0.450 min retention time window of the same peak in other samples. Peak areas and retention times were processed further in Microsoft Excel. Different HPLC-MS methods were used for HMOs and MUC2 *O*-glycans, although in both instances chromatographic resolution was achieved using a Hypercarb (Thermo Scientific) PGC column (100 mm × 2.1 mm; particle size 3 μm).

#### HMOs

To avoid resolving between α- and β-HMO-anomers by PGC HPLC, lyophilized samples were reduced by treatment with freshly prepared 1 M NaBH_4_ in 50 mM NH_4_OH at 60 °C prior to analysis. After 2 h, reactions were quenched and the reduced HMO alditols were desalted by ENVICarb SPE exactly as described above for MUC2-derived glycans. To maintain consistency with independently analyzed cross-sectional (22) and longitudinal data sets (Table 2), identical HPLC-MS conditions were maintained, albeit different HPLC columns were used for each sample set (and insufficient sample was available to perform a direct head-to-head comparison); essentially identical conditions have also been reported previously by other groups (43, 46). Although ISTD-corrected abundances for eight known HMOs (and 15 putative sulfated HMOs) have been previously reported for the compositional data set, these data were completely reanalyzed with an expanded list of 21 HMO standards as well as formulas consistent with dozens of additional sulfate/GlcA-containing HMOs that were not included in prior analyses (Fig. S2 and Table S1). The following HPLC gradient conditions were used with mobile phase A being 0.1% formic acid and B ACN + 0.1% formic acid: 0 to 5 min (0–5% B), 5 to 15 min (5–20% B), 15 to 20 min (20–40% B), 20 to 25 min (40–80% B), 25 to 27 min (isocratic at 80% B), and 27.0 to 27.1 min (80–5% B). The flow rate was maintained at 250 μl/min. Representative HRMS spectra for novel GlcA/sulfate-containing HMOs (Fig. S4) were generated by exporting MassHunter data as text files that were subsequently plotted using GraphPad Prism (Version 10.4.1); mass errors (δ; in ppm) for the molecular ion ([M-H]^-^ peak) and the theoretical relative abundances of the isotopic distributions of isotopologues were calculated using MassHunter.

#### O-glycans

Desalted *O*-glycans released by reductive β-elimination were resolved by PGC and detected by HRMS exactly as previously described (37). Mobile phases A and B were 10 mM ammonium bicarbonate (A) and 80% ACN containing 10 mM ammonium bicarbonate (B), and a flow rate of 0.45 ml/min was maintained. The following gradient was used: 0 to 15 min (0–15% B), 15 to 22.5 min (15–25% B), 22.5 to 25 min (25–40% B), 25 to 25.2 min (40–98% B), 22.5 to 25 min (isocratic at 98% B), after which the column as washed for 3 min in phase A prior to the next injection. HRMS parameters for HMOs and *O*-glycans were identical.

#### Tandem MS (MS/MS)

Data-dependent acquisition was used to produce product ion spectra for both HMOs and MUC2-*O*-glycans; in both cases, the maximum number of hydrogens was limited to one, ensuring only [M-H]^-^ peaks were selected for fragmentation, and an upper limit of 50 ppm was selected for mass accuracy. For HMO analyses, a single pooled sample was made by combining a small portion of each sample, both stool and milk-derived, from the longitudinal sample set. This pool was used to obtain MS/MS spectra for all HMOs based on an inclusion list of all formulas containing sulfate, GlcA, or both; known HMOs, with the exception of LST isobars, were excluded. Collision energies for HMOs were linearly increased with *m*/*z* from *m*/*z* 300 (25 V) to 1500 (60 V). The collision energies for *O*-glycans were similarly linearly scaled to *m*/*z* from *m*/*z* 300 (30 V) to 1500 (50 V) with the following exceptions that were determined empirically: 10010_2 (30 V), 32100_1, and _5 (45 V), 32100_2, _3, _4, and _6 (40 V), 22000_3 (37 V), 22010_1 (45 V), 22110_1 (50 V), 21001_1 (35 V), and 21000_4 (35 V). MUC2-*O*-glycans were not pooled for MS/MS analyses. For the structural assignment of HMOs, it was assumed that all structures contained a reduced lactose (*i.e.*, Hex2) core; likewise, all *O*-glycans were assumed to contain a reduced GalNAc (*i.e.*, HexNAc) at their reducing end. Putative MUC2 *O*-glycan structures were assigned with the assistance of GlycoWorkbench 2.1 (73) informed by prior structural analyses of human MUC2 (7, 8, 9). The nomenclature recommended by Domon and Costello was used to annotate product ion spectra (74). All product ion spectra displayed herein (Figs. 4, 2*B*, S5, S10, and S15) were produced as line graphs from the original MassHunter data using GraphPad Prism with *m*/*z* 160 as the low mass cutoff.

### Enzymatic synthesis of GlcA- and sulfate-containing HMOs

Recombinant human glycosyl-transferases were used to demonstrate the biochemical feasibility of producing GlcA-, and/or Fuc-containing HMOs from known analogues. These enzymes were used exactly according to the manufacturer’s instructions, beginning with 10 nmol of each HMO acceptor. Half of each sample was analyzed by CE-LIF after drying and labelling with APTS without SPE. The other half was dried, reduced to the corresponding alditol exactly as described above, and analyzed by HPLC-MS or MS/MS after ENVICarb clean-up.

### Statistical procedures

All post-acquisition (CE-LIF or HPLC-MS) data processing was performed in Excel (Microsoft). Statistical procedures and the Spearman correlation analyses were performed using GraphPad Prism. Data normality for the cross-sectional HMO data (HPLC-MS) was assessed using a Shapiro-Wilk test (GraphPad). PLS-DA with the entire MUC2-*O*-glycan data set classified based on the four donors was performed using Metaboanalyst 6.0 (www.metaboanalyst.ca/) with one-fifth of the minimum value imputed for non-detected glycans in instances in which a specific glycan was positively identified in over half of the samples. A similar imputation strategy was performed (in Excel) before statistical analysis of the cross-sectional HMO data (HPLC-MS); no other data were imputed. *p* values < 0.05 were considered to be statistically significant.

## Data availability

In addition to the data contained within the manuscript and its supporting information files, all new HPLC-MS and tandem MS data have been deposited to the publicly accessible repository *Open Science Framework (OSF)* with the project ID Vd89r. Previously collected data that has been completely reanalyzed herein may be accessed from the OSF repository with project ID 2xvgh.

## Supporting information

This article contains supporting information.

## Conflict of interest

The authors declare that they have no conflicts of interest with the contents of this article.

## Supporting information

Supporting Figures and References
