# Supplementary material for: Longitudinal analysis of human milk oligosaccharides and mucin-2-glycans in infants reveals enrichment of sulfated and glucuronic acid-bearing HMOs
Source: J Biol Chem. 2026 Apr 28;302(6):113099. doi: 10.1016/j.jbc.2026.113099 (PMC13241717; doi:10.1016/j.jbc.2026.113099)
Supplement: Supporting Figures and References [file mmc1.docx]

**SUPPORTING INFORMATION**

**Longitudinal analysis of human milk oligosaccharides (HMOs) and Mucin-2 *O*-glycans in the infant gut reveals enrichment of sulfated and glucuronic acid-containing HMOs**

Nitin^1^, Parandis Daneshgar^1^, Darrek Kniffen^2^, Sara D. Vicaretti^1^, Brandon Whitmore^2^, Linus Kipchumba^2^, Candice Quin^2^, Deanna L. Gibson^2,3^, Kirk S. B. Bergstrom^2^, and Wesley F. Zandberg^1,3^

The University of British Columbia, Departments of ^1^Chemistry, ^2^Biochemistry and Molecular Biology, and ^3^Biology, Kelowna, BC, V1V 1V7, Canada

**Contents Page**

**Figure S1**. Neonatal GI glycomics..…………………………..…………………………………………...2

**Figure S2.** List of HMO standards……………………………..………….………………………………3

**Table S1.** Relative abundances of known and putative HMOs quantitated by HPLC-MS: Cross sectional data set (5 months; N = 16)……………………………………………………………………...4

**Figure S3.** Longitudinal analysis of relative HMO abundances in milk and stool: CE-LIF……………5

**Table S2.** List of all novel sulfate- and GlcA-containing HMOs found in stool and milk samples: Longitudinal data set…………………………………………………………………………………….….6

**Figure S4.** HRMS spectra for unique sulfate-, GlcA-, and/or Neu5Ac-containing HMOs…………….7

**Figure S5.** Tandem MS spectra of novel sulfated HMOs………………………………………………..8

**Figure S6.** Longitudinal analysis of relative HMO concentrations in milk and stool: HPLC-MS……9

**Figure S7.** MUC2 purification and lectin blotting…………………………………………………..,......10

**Figure S8.** Longitudinal analysis of relative abundances of MUC2 glycan classes…………………11

**Figure S9.** Partial least squares discriminate analysis (PLS DA) of the neonatal MUC2 glycome...12

**Figure S10.** Product ion spectra for selected MUC2-derived O-glycans……………………………..13

**Figure S11.** Spearman correlation analysis……………………………………………………….....…16

**Figure S12.** Heat maps depicting associations between HMOs and MUC2 glycans………...……..17

**Figure S13.** Lactose-priming hypothesis………………………………………………………………..18

**Figure S14.** CE-LIF and HPLC-MS evidence for the biosynthesis of GAG-primer-like HMOs……..20

**References…**……………………………………………………………………………………………...21


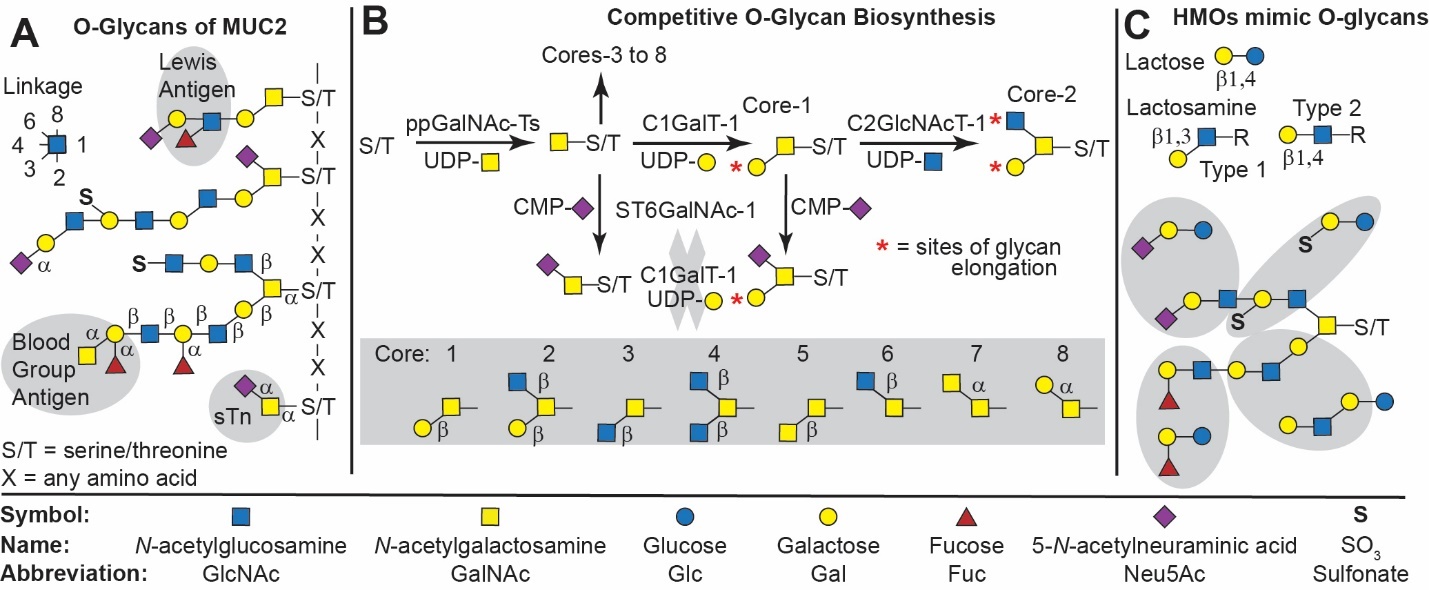


**Figure S1. Neonatal gastrointestinal (GI) glycomics. (A)** The GI tract is protected by mucus, a substance composed of large glycoproteins called mucins. Mucin-2 (MUC2), the major mucin protecting the colon, is characterized by thousands of serine/threonine (S/T)-linked glycans attributable to 80 percent of its mass. As these glycans are covalently linked through the S/T hydroxyl (OH) groups, thet are dubbed *O*-glycans. Four hypothetical *O*-glycans are depicted in Consortium for Functional Glycomics nomenclature where in different symbols/colors depict monosaccharides and the regiochemistry of each glycosidic linkage is denoted by the angle of the line between residues; the stereochemical assignment (α or β) of the most commonly observed linkages are denoted. The vertical line between S/T and other amino acids (X) indicates the peptide bonds of the invariant polypeptide MUC2 backbone. In addition to serving as an energy source for the colon microbiota, MUC2-borne *O*-glycans contain glyco-epitopes with known immunological activities such as Lewis (Le; sialyl-Le^X^ is shown) or blood group (A is shown) antigens. Truncated glycans like the sialyl-Tn antigen (sTn) are frequently associated with inflammation. **(B)** The competitive glycosyltransferases (GTs) in the MUC2 glycan biosynthetic pathways yield hundreds of dynamic structures that endow it with different functional properties. GTs catalyze glycoside bond-formation between acceptor substrates and nucleotide-linked donors, uridine diphosphate (UDP) in the case of GlcNAc, GalNAc and Gal, and cytosine monophosphate (CMP) or guanosine diphosphate (GDP) for Neu5Ac and Fuc, respectively. *O*-glycosylation is initiated by one of >21 polypeptide-GalNAc-transferases (ppGalNAc-Ts), forming a S/T-α-GalNAc that may be elaborated by one or more GT(s) creating up to eight known *O*-glycan cores. Core-1 Gal-transferase-1 (C1GalT-1) and core-2 GlcNAc-transferase-1 (C2GlcNAcT-1) initiate the formation of core-2 glycans. Note that C2GlcNAcT-1 necessarily blocks the activity of a core-GalNAc-modifying sialyltransferase (ST6GalNAc-1) and that ST6GalNAc-1 action before C1GalT-1 yields sTn, a glycan that cannot be elongated by other GTs capable of installing type 1 or type 2 polylactosamine chains that are in turn further elaborated with additional Fuc, Neu5Ac or sulfonate (SO_3_) moieties. **(C)** Soluble human milk oligosaccharides (HMOs) share a lactose core modified by GTs in pathways similar to those yielding MUC2; four examples are shown. Since HMOs reach the colon intact (*i.e.* undigested), they may likewise interact with the colon microbiota and thus compete with MUC2-borne glycans in terms of microbial metabolism and/or adhesion or fulfill other immunological functions.


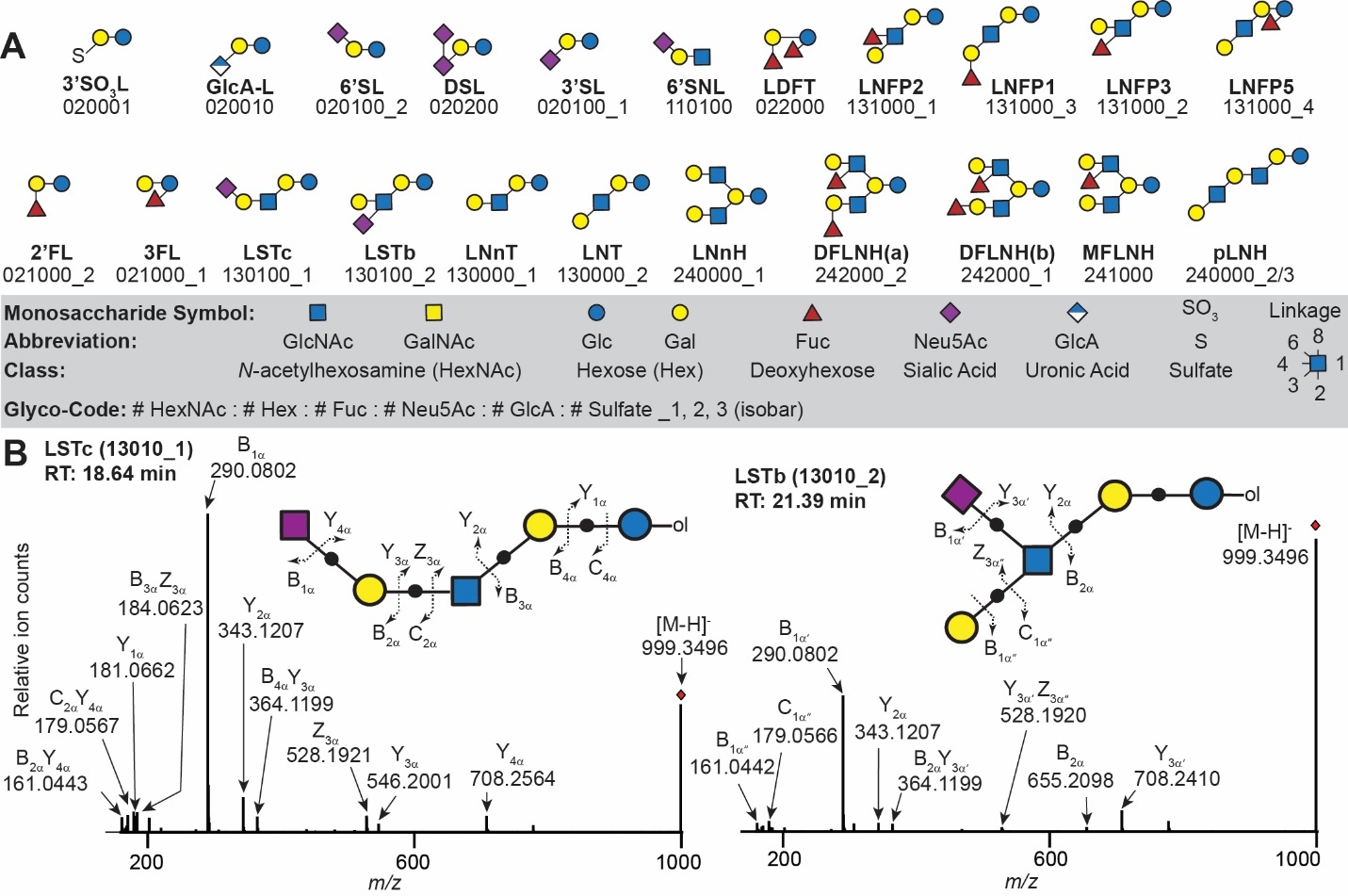
_­_

**Figure S2. HMO standards used for CE-LIF and HPLC-MS annotations. (A)** Commercially-available HMO (or putative HMO) standards depicted in Consortium for Functional Glycomics nomenclature. All HMOs or other glycans (such as mucin-derived *O*-glycans) may also be represented with seven-digit glyco-codes where each of the first six digits denotes the number of *m*/*z*-distinct HexNAc, Hex, Fuc, Neu5Ac, GlcA, and sulfate residues, respectively and the _1, _2, *etc.* suffix denotes the number of HPLC-resolved isobars. To determine chemical formulas from GlycoCodes, add C_8_H_13_N_1_O_5_, C_6_H_10_O_5_, C_6_H_10_O4, C_11_H_17_N_1_O_8_, C_6_H_8_O_6_, and SO_3_ for each non-reducing HexNAc, Hex, Fuc, Neu5Ac, GlcA, or sulfate residue, respectively, and either C_8_H_17_N_1_O_6_ or C_6_H_14_O_6_ for a reducing end HexNAc (as in an *O*-glycan) or Hex (as in an HMO). **(B)** The LSTc standard was deduced to be a mixture of two isobars, assigned as LSTc and LSTb on the basis of tandem MS data. The “ol” on the reducing end Glc of LSTc or LSTb indicates that these HMOs have been reduced to their corresponding alditols prior to analysis. The small black circles between monosaccharide residues denotes the interglycosidic oxygen atoms. The fragmentation nomenclature used is that recommended by Domon and Costello.

**Table S1. Abundances of HMOs quantitated by HPLC-MS: Cross sectional data set^(a)^**

| **HMO^(b)^** | **RT (SD); min^(c)^** | **Relative Abundance; % Total^(d)(e)^** | | **P^(f)^** |
| --- | --- | --- | --- | --- |
|  |  | **Se+ (N)** | **Se- (N)** |  |
| 2’FL | 10.7 (0.78) | 9.47 (12) | 0.19 (4) | 0.002 |
| 3FL | 6.5 (0.11) | 0.39 (11) | 0.62 (4) | 0.042 |
| 3’SL | 18.9 (1.80) | 12.09 (12) | 13.51 (4) | 0.521 |
| 6’SL | 13.2 (1.36) | 3.02 (12) | 8.04 (4) | 0.058 |
| 6’SLN | 13.3 (1.31) | 0.02 (2) | 0.06 (2) | 0.333 |
| LDFT | 11.9 (0.09) | 5.99 (12) | 0.04 (4) | 0.001 |
| LNT | 11.3 (0.67) | 19.76 (12) | 23.16 (4) | 0.020 |
| LNnT | 11.5 (0.09) | 7.72 (4) | (0) |  |
| LNFP1 | 10.9 (0.75) | 13.64 (12) | 0.032 (4) | 0.001 |
| LNFP2 | 9.2 (0.93) | 15.31 (12) | 22.64 (4) | 0.261 |
| LNFP3 | 9.5 (4.00) | 0.42 (7) | 3.74 (3) | 0.261 |
| LNFP5 | 11.2 (0.08) | 1.67 (12) | 4.46 (4) | 0.001 |
| LSTc | 17.4 (0.97) | 0.98 (12) | 1.13 (4) | 0.770 |
| LSTb | 18.5 (1.14) | 2.51 (10) | 3.26 (3) | 0.683 |
| LNnH | 13.2 (0.07) | 0.55 (12) | 0.36 (4) | 0.261 |
| pLNH | 22.6 (0.08) | 1.42 (12) | 0.71 (3) | 0.103 |
| DFLNH(a) | 11.4 (1.12) | 1.18 (12) | < 0.01 (2) | 0.001 |
| DFLNH(b) | 10.5 (2.05) | 1.47 (12) | 9.14 (4) | 0.002 |
| MFLNH3 | 11.1 (0.66) | 2.57 (11) | 0.76 (3) | 0.175 |
| DFpLNH2 | 9.7 (0.08) | 0.85 (12) | 0.71 (4) | 0.953 |
| **Putative Sulfated HMOs^(g)^** | | | | |
| 020001_1 | 12.6 (0.78) | 0.61 (2) | 1.01 (1) |  |
| 020001_2 | 14.5 (1.36) | < 0.01 (7) | 0.69 (2) | 0.261 |
| 020001_3 | 27.3 (2.23) | 0.62 (3) | 0.84 (1) |  |
| 021001 | 20.2 (1.56) | 0.12 (8) | 0.12 (0) | 0.379 |
| 131001 | 15.7 (1.55) | 0.09 (11) | 0.11 (4) | 0.521 |
| 240001 | 15.2 (0.08) | 0.08 (8) | 0.09 (2) | 0.599 |
| 241001 | 17.0 (1.11) | 1.11 (10) | 0.03 (4) | 0.133 |
| 242001 | 17.0 (3.83) | 3.83 (1) | 0.07 (0) | 0.683 |
| 243001 | 15.4 (1.37) | 1.37 (9) | 0.07 (0) | 0.173 |

**Notes: (a)** All milk samples were collected at five-months post-partum; *N* = 12 Se+ and 4 Se-. **(b)** HMO identities were established based on *m*/*z* and retention time-match to commercially-available standards as listed in Supporting Figure S2. **(c)** RT = retention time; SD = standard deviation. **(d)** N denotes the number of samples in each secretor group wherein an HMO was unambiguously identified. **(e)** One fifth the minimum area of an HMO was imputed for missing values in instances where it was positively identified in over half (*i.e.* eight or more) of samples. Imputation was performed before data normalization. **(f)** Two-tailed Mann-Whitney U-Test; *P* < 0.05 was considered to be significant. **(g)** Glyco-codes are assigned exactly as described in Supporting Figure S2 with the first six digits denoting the number of hexosamine, hexose, fucose, Neu5Ac, GlcA, and sulfate residues, respectively, and the _suffix denoting the number of HPLC-resolvable isobars.


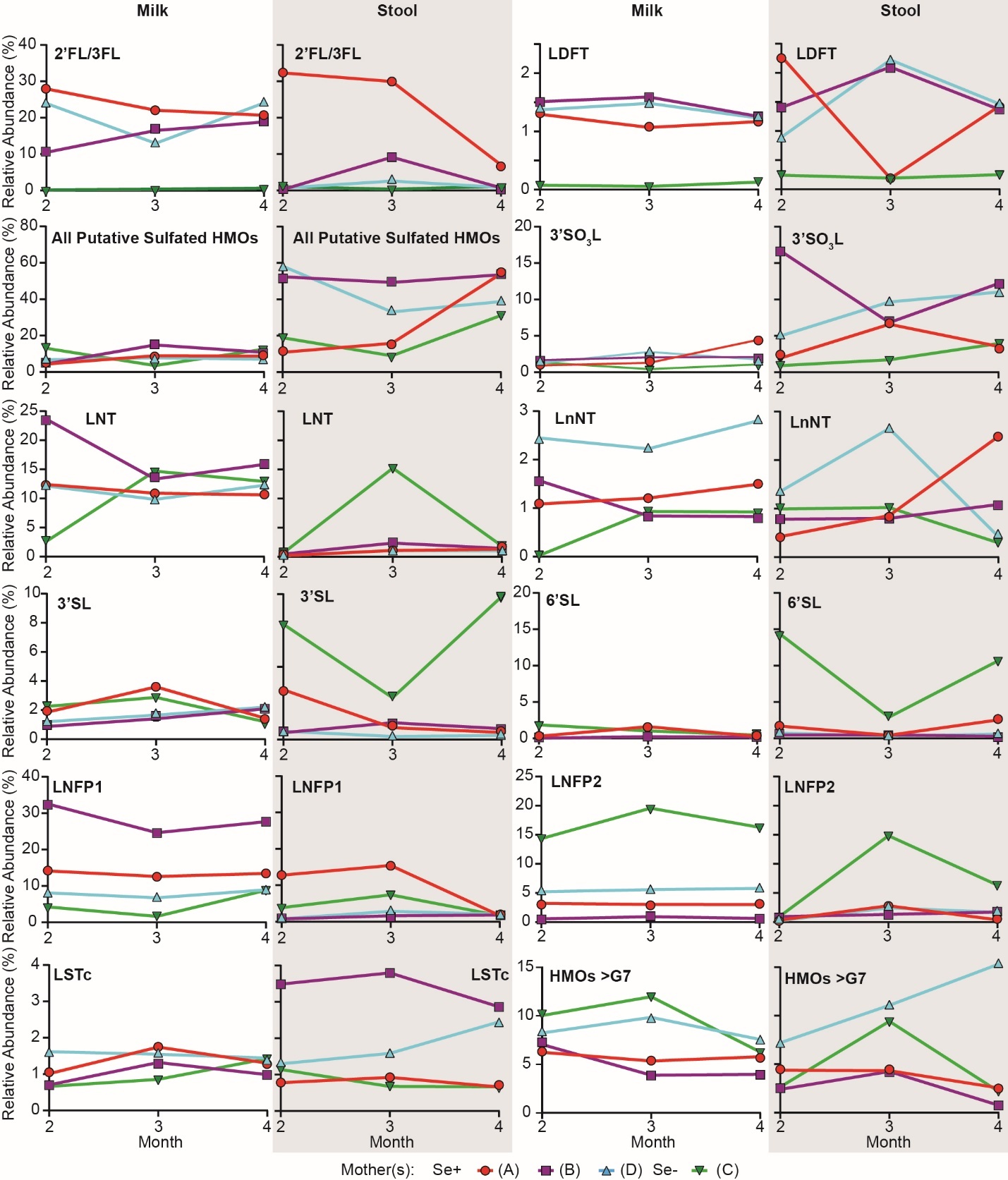


**Figure S3. Longitudinal analysis of milk or stool HMOs by CE-LIF in mother-infant pairs reveals no obvious temporal trends in relative concentrations of selected HMOs except enrichment or depletion in stool.** Note that HMO concentrations in milk *vs.* stool are plotted on identical y-axes. “>G7” denotes HMOs with CE mobilities exceeding that of the internal standard, maltoheptaose (*i.e.* ~ 7 glucose units; G7). Migration times of these large glycans were so variable that they could not be consistently integrated; therefore, all peak areas from G7 to the end of each CE run (12 min) were summed and are reported in aggregate as are putative sulfated HMOs.

**Table S2. List of all novel sulfate- and GlcA-containing HMOs found in stool and milk samples: Longitudinal data set^(a)^.**

| **HMO/Code** | **RT(min)^(b)^** | **N (milk)** | **N (stool)** | **HMO/Code** | **RT (min)** | **N (milk)** | **N (stool)** |
| --- | --- | --- | --- | --- | --- | --- | --- |
| **HMOs identified with standards** | | | | **Sulfate-containing HMOs; continued** | | | |
| 2’FL | 10.7 | 15 | 15 | 021001_2 | 11.6 | 6 | 2 |
| 3FL | 5.9 | 14 | 10 | 130001_1 | 21.8 | 8 | 7 |
| 3’SL | 20.8 | 15 | 5 | 130001_2 | 17.1 | 9 | 6 |
| 6’SLN | 13.5 | 15 | 0 | 130001_3 | 23.2 | 2 | 3 |
| LDFT | 12.1 | 13 | 14 | 130001_4 | 20.5 | 0 | 3 |
| LNT | 11.8 | 15 | 14 | 131001_1 | 20.5 | 10 | 1 |
| LNnT | 12.1 | 10 | 13 | 131001_2 | 21.0 | 6 | 2 |
| LNFP1 | 11.4 | 15 | 14 | 312001 | 12.6 | 4 | 0 |
| LNFP2 | 9.6 | 15 | 12 | 241001_1 | 19.3 | 11 | 3 |
| LNFP3 | 9.8 | 15 | 11 | 241001_2 | 21.6 | 4 | 2 |
| LNFP5 | 11.7 | 15 | 10 | 242001_1 | 19.1 | 10 | 5 |
| LSTc | 19.0 | 15 | 9 | 242001_2 | 17.8 | 10 | 0 |
| LSTb | 20.4 | 9 | 12 | 242001_3 | 16.9 | 4 | 5 |
| LNnH | 14.0 | 15 | 10 | 242001_4 | 19.9 | 4 | 4 |
| pLNH_1 | 14.1 | 15 | 6 | 243001_1 | 17.8 | 7 | 1 |
| pLNH_2 | 15.9 | 15 | 10 | **GlcA- and sulfate-containing HMOs** | | | |
| DFLNH(a) | 12.4 | 15 | 14 | 020011_1 | 9.9 | 3 | 0 |
| DFLNH(b) | 11.4 | 15 | 14 | 020011_2 | 8.2 | 0 | 3 |
| MFLNH3 | 12.9 | 15 | 13 | 021011 | 4.9 | 4 | 1 |
| DFpLNH2 | 10.7 | 11 | 12 | 030011_1 | 12.0 | 15 | 8 |
| **GlcA-containing HMOs^(c)^** | | | | 030011_2 | 12.4 | 10 | 4 |
| GlcA-L | 13.3 | 11 | 8 | 030011_3 | 13.3 | 11 | 0 |
| 011010_1 | 12.5 | 6 | 0 | 211011 | 10.7 | 4 | 0 |
| 011010_2 | 10.8 | 3 | 0 | 030111_1 | 20.1 | 5 | 2 |
| 110010 | 9.2 | 4 | 0 | 030111_2 | 20.8 | 13 | 3 |
| 021010_1 | 4.9 | 10 | 4 | 030111_3 | 21.7 | 7 | 4 |
| 021010_2 | 16.2 | 0 | 7 | 240001 | 10.2 | 5 | 1 |
| 200010 | 15.8 | 5 | 3 | 031111_1 | 18.0 | 8 | 0 |
| 111010 | 13.8 | 5 | 1 | 031111_2 | 18.1 | 14 | 1 |
| **Sulfate-containing HMOs** | | | | 031111_3 | 18.7 | 8 | 3 |
| 021001_1 | 24.9 | 3 | 0 | 031111_4 | 19.6 | 2 | 4 |

**Notes: (a)** Samples from N = 3 separate Se+ mother-infant pairs were collected at 2-, 3-, 4-, and 5-months *post-partum*; N = 1 Se- mother-infant pair was collected at 2, 3, and 4 months. Thus, 15 paired samples were analyzed. **(b)** RT = retention time. **(c)** Glyco-codes are assigned exactly as described in Supporting Figure S2 with the first six digits denoting the number of hexosamine, hexose, fucose, Neu5Ac, GlcA, and sulfate residues, respectively, and the _suffix denoting the number of HPLC-resolvable isobars.


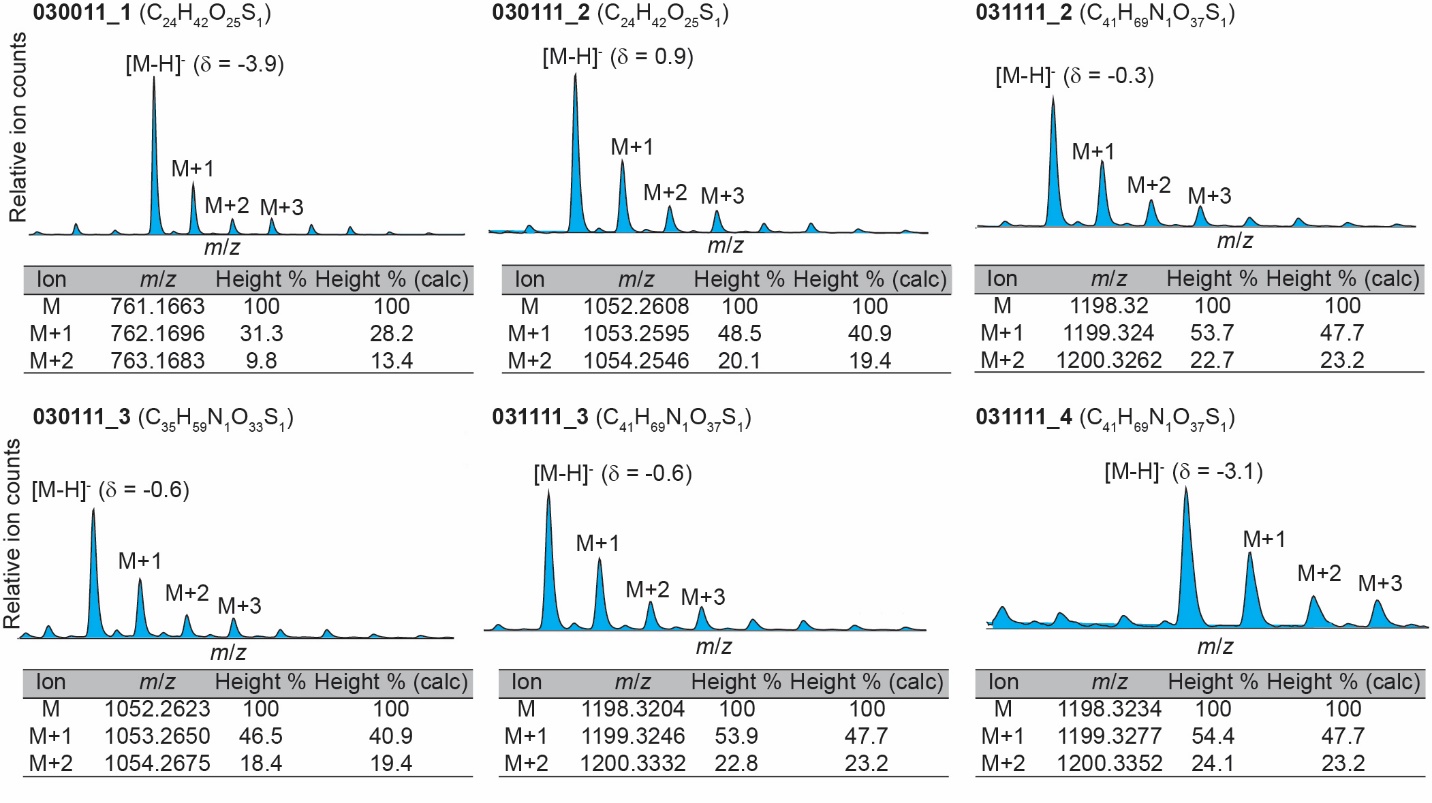


**Figure S4. HRMS spectra for novel GlcA-, sulfate-, Neu5Ac, and/or Fuc-containing HMOs.** Mass spectra for ions with formulas consistent with rare combinations of monosaccharides—namely, hexoses (presumably Glc and Gal), GlcA, Neu5Ac, and, in some instances, Fuc—plus sulfate residues are depicted. The mass error for the molecular ions are noted along with peak heights (relative to the [M-H]^-^ molecular ion) for isotopologue peaks that could not be resolved by QToF MS. Note that the high relative abundance of [M+2-H]^-^ peaks is, along with the HRMS *m*/*z*, evidence of reasonably abundant (4.2%) ^34^S isotopes. Glyco-codes are assigned exactly as described in Supporting Figure S2 with the first six digits denoting the number of hexosamine, hexose, fucose, Neu5Ac, GlcA, and sulfate residues, respectively, and the _suffix denoting the number of HPLC-resolvable isobars. Retention times and frequency of detection of each HMO are reported in Supporting Table S2.


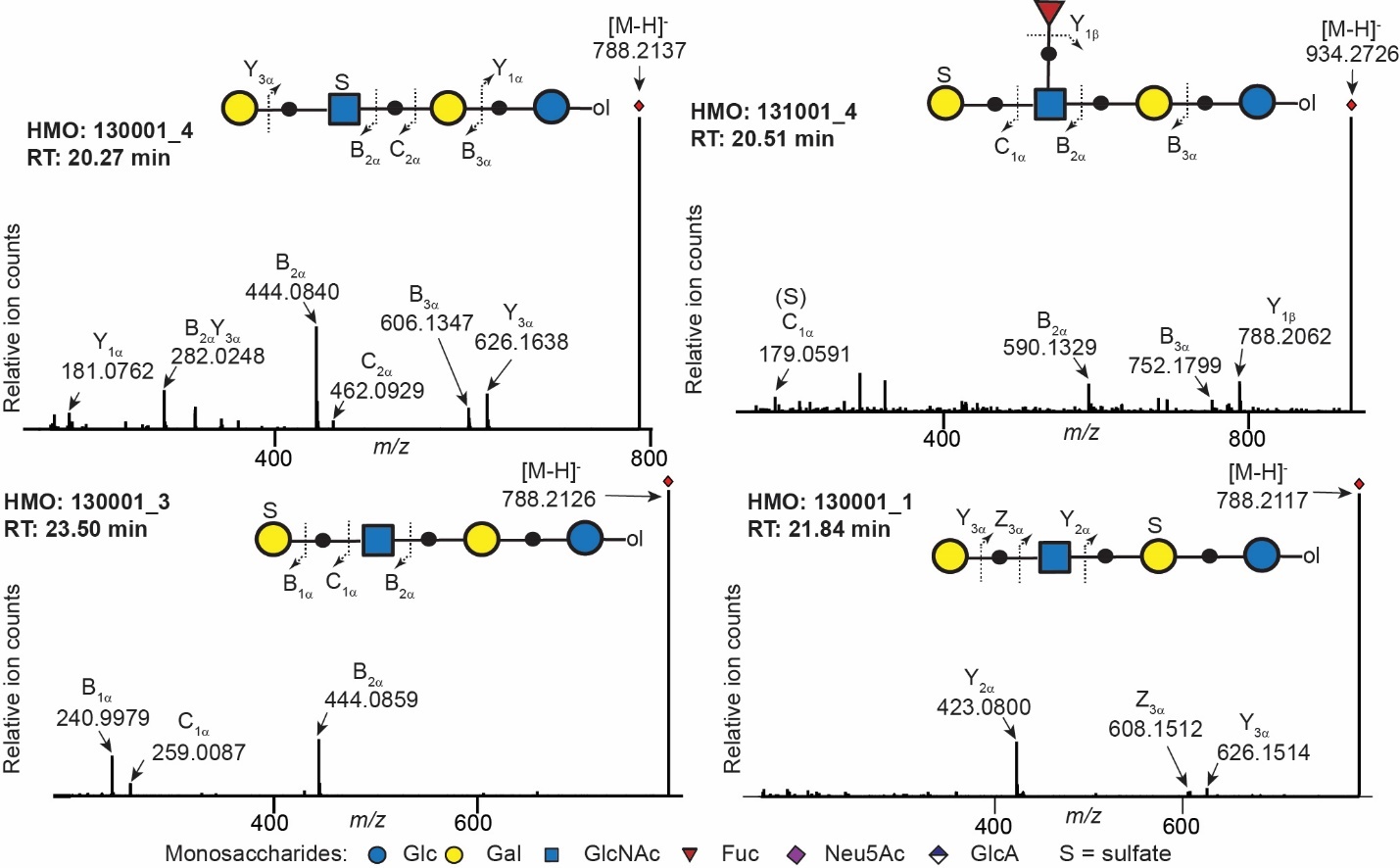


**Figure S5. Putative structures of four novel sulfated HMOs deduced by tandem MS.**


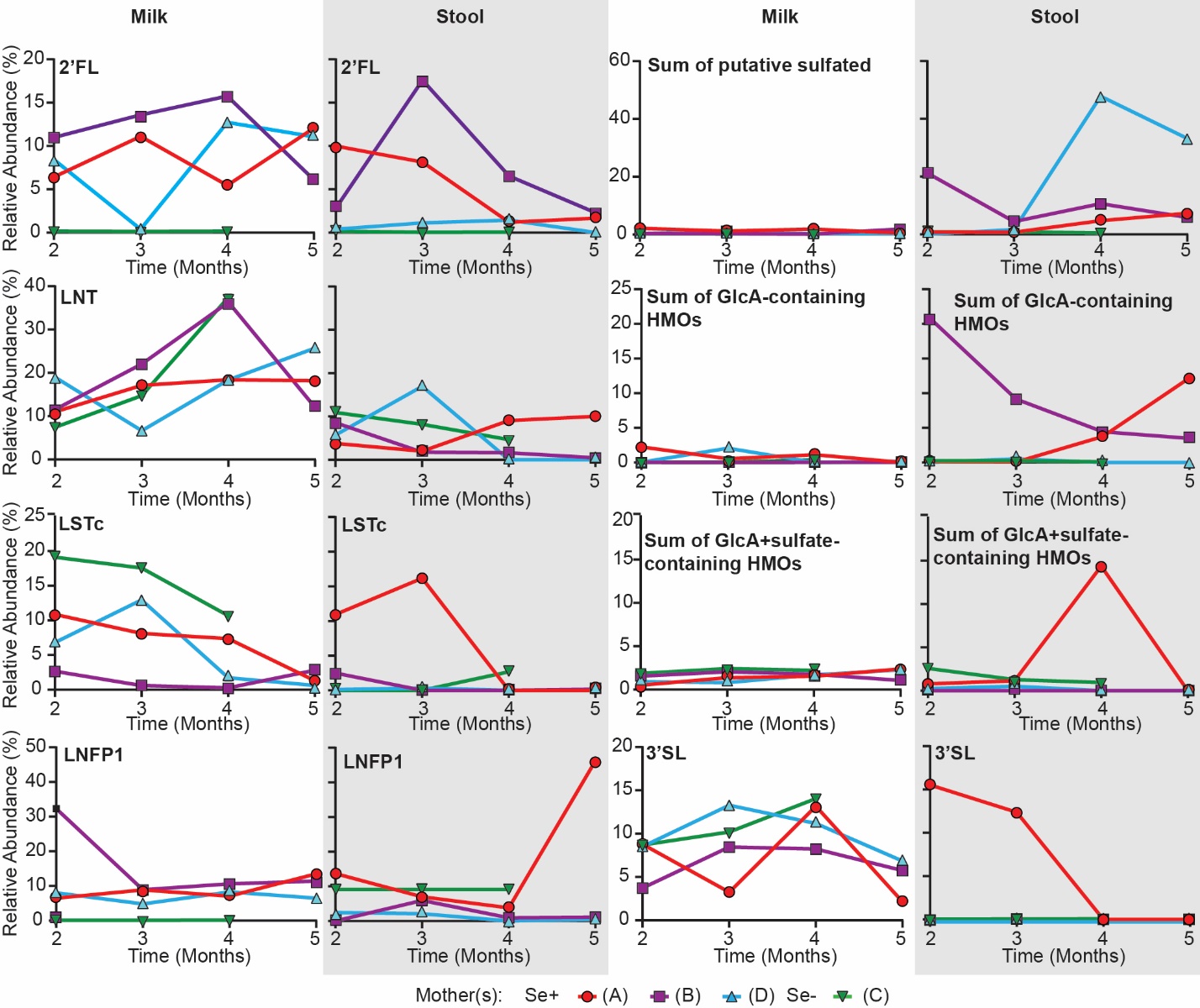


**Figure S6. Longitudinal HPLC-MS analysis HMO relative concentrations in paired milk and stool samples.** Relative abundances of selected HMOs or HMO classes are depicted. Line plots for each HMO abundance in milk and stool are plotted on the same y-axes. HMO abundances were expressed as a percentage of 21 HMOs verified with standards (2’FL, 3FL, 3’SL, 6’SNL, GlcA-L, LDFT, LNT, LNnT, LNFP1, LNFP2, LNFP3, LNFP5, LSTb, LSTc, LNnH, pLNH (two isobars), MFLNH3, DFLNH(a), DFLNH(b), and DFpLNH2) as well as 39 putative GlcA- and sulfate-containing HMOs. Note that for these line plots specific HMOs that were not detected at a given time point were imputed as zeros.


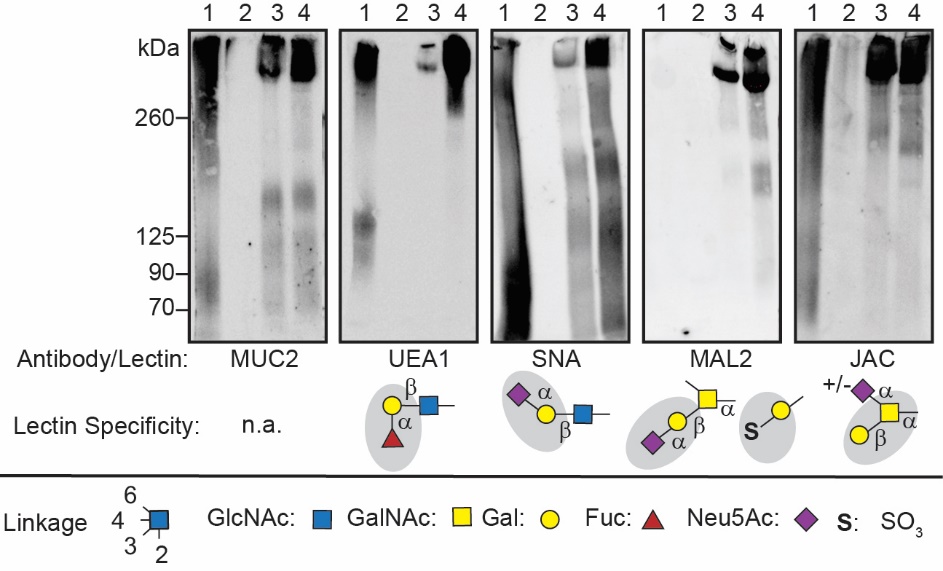


**Figure S7. MUC2 purity analysis revealed by composite gel electrophoresis follow by immuno- or lectin-blotting.** Equivalent amounts of sample were loaded for all blots. Lanes: 1 = porcine stomach mucin (positive control), 2 = bovine serum albumin (negative control), 3 = MUC2 from donor B, 4 = MUC2 from donor D.


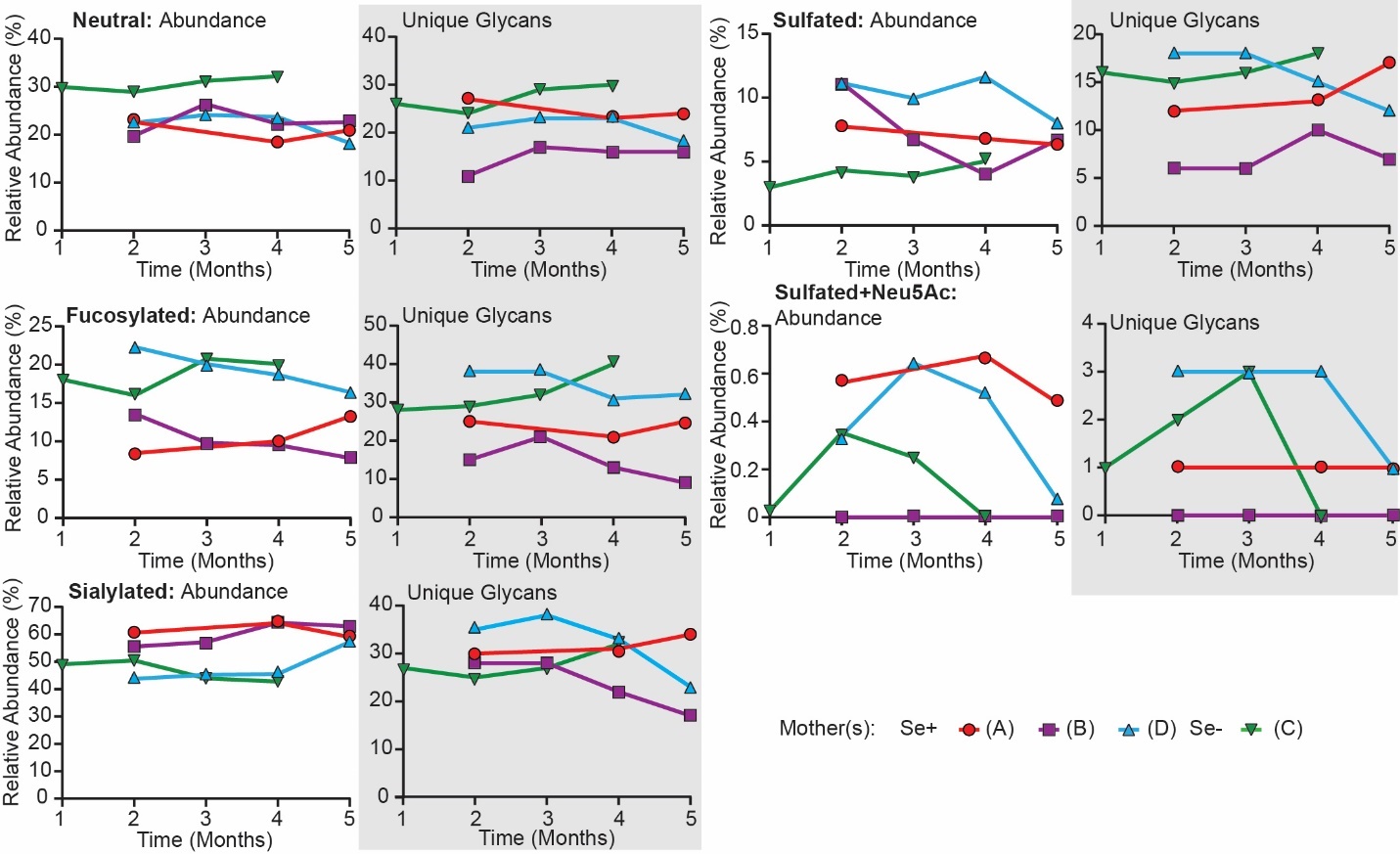


**Figure S8. Longitudinal analysis of MUC2 glycan classes in four exclusively breast-fed infants.** Putative *O*-glycans, derived from stool-associated MUC2, were assigned into structurally-relevant classes, namely neutral (only HexNAc and Hex residues), fucosylated (HexNAc, Hex, and Fuc), sialylated (HexNAc, Hex, and/or Fuc, and Neu5Ac) or sulfated+Neu5Ac (wherein both Neu5Ac and sulfate moieties were present). Both relative abundances and number of unique glycans in each class are plotted as a function of the number of months post-partum. Note that insufficient stool sample from month 3 for mother/infant pair “A” was available for MUC2 glycomics.


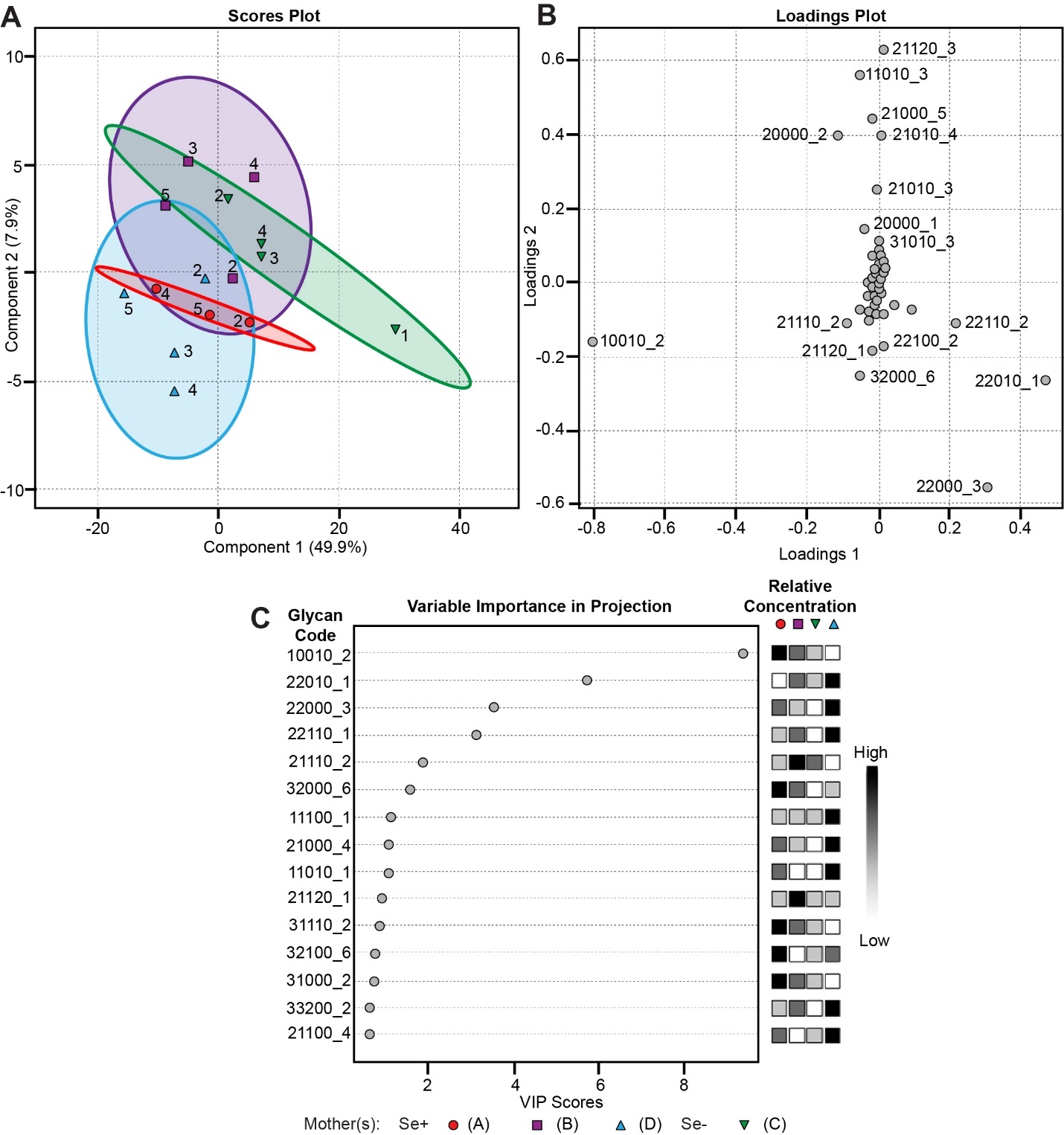


**Figure S9. Partial least squares discriminate analysis indicates that four glycans contribute to the majority of the discrimination between MUC2 samples collected from neonatal stool samples.** **(A)** Scores plot for the first two components explaining 52.6% of the variability between the four donor groups. The individual shapes denote each individual sample while the Arabic numerals in the score plot denotes the month *post-partum* of stool sample collection. The red, purple, blue and green ellipses denote donors A, B, C, and D, respectively with the shade area denoting the 95% confidence interval. **(B)** The loadings plot indicates that 10010_2 and 22010_1 contribute to separation along component 1 while 21120_3 and 22000_3 most significantly impact component 2. **(C)** Variable importance in projection scores identify four glycans that account for the majority of the discrimination between the MUC2 glycomes. As in Figure S2, glycans are represented by glyco-codes where each of the first digits denotes the number of *m*/*z*-distinct HexNAc, Hex, Fuc, Neu5Ac, and sulfate residues, respectively, and the _1, _2, *etc.* suffix denotes the number of HPLC-resolved isobars. In contrast with HMOs, all putative *O*-glycans were assumed to contain a reduced GalNAc (*i.e.* an alditol) at their reducing ends.


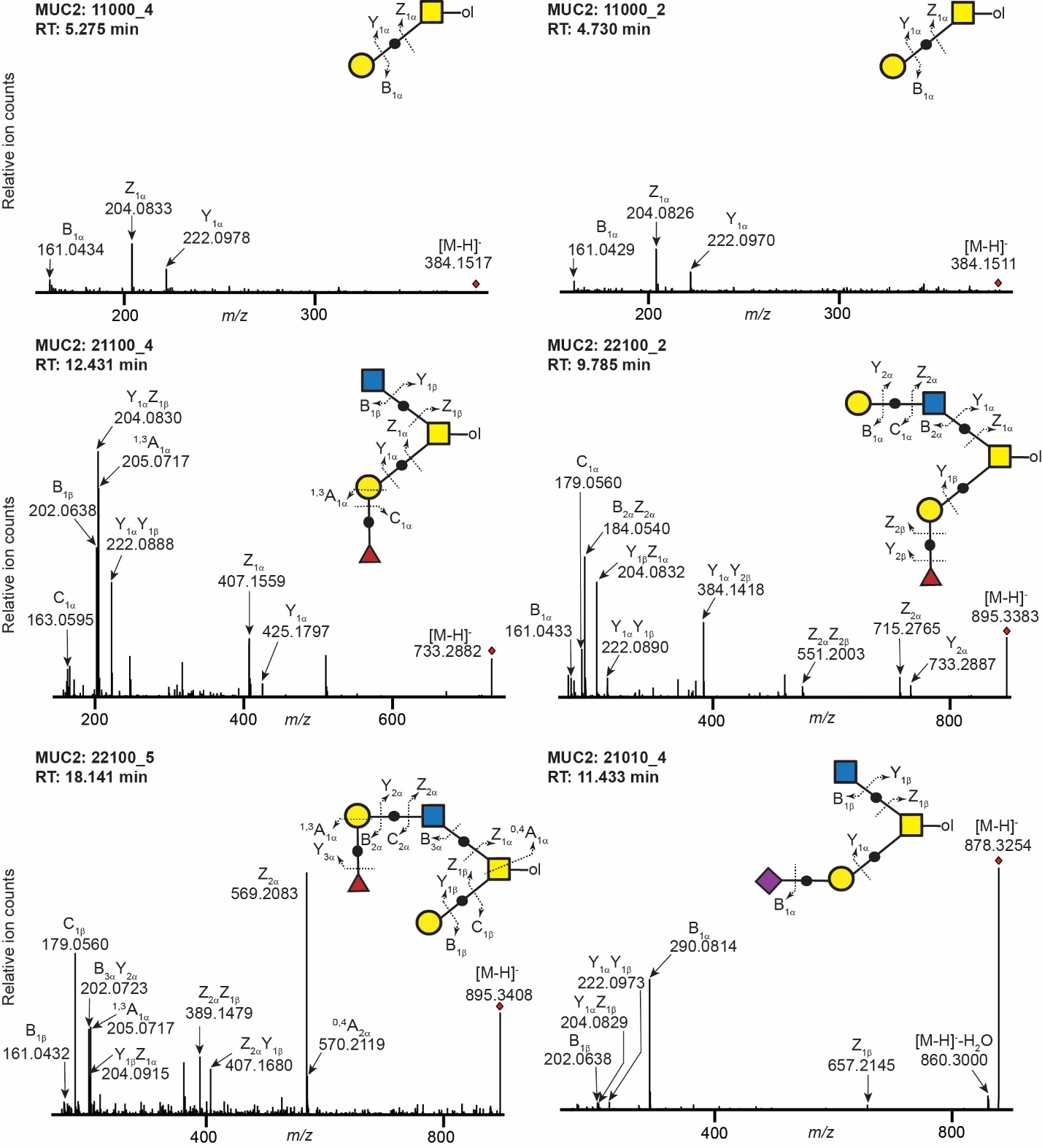


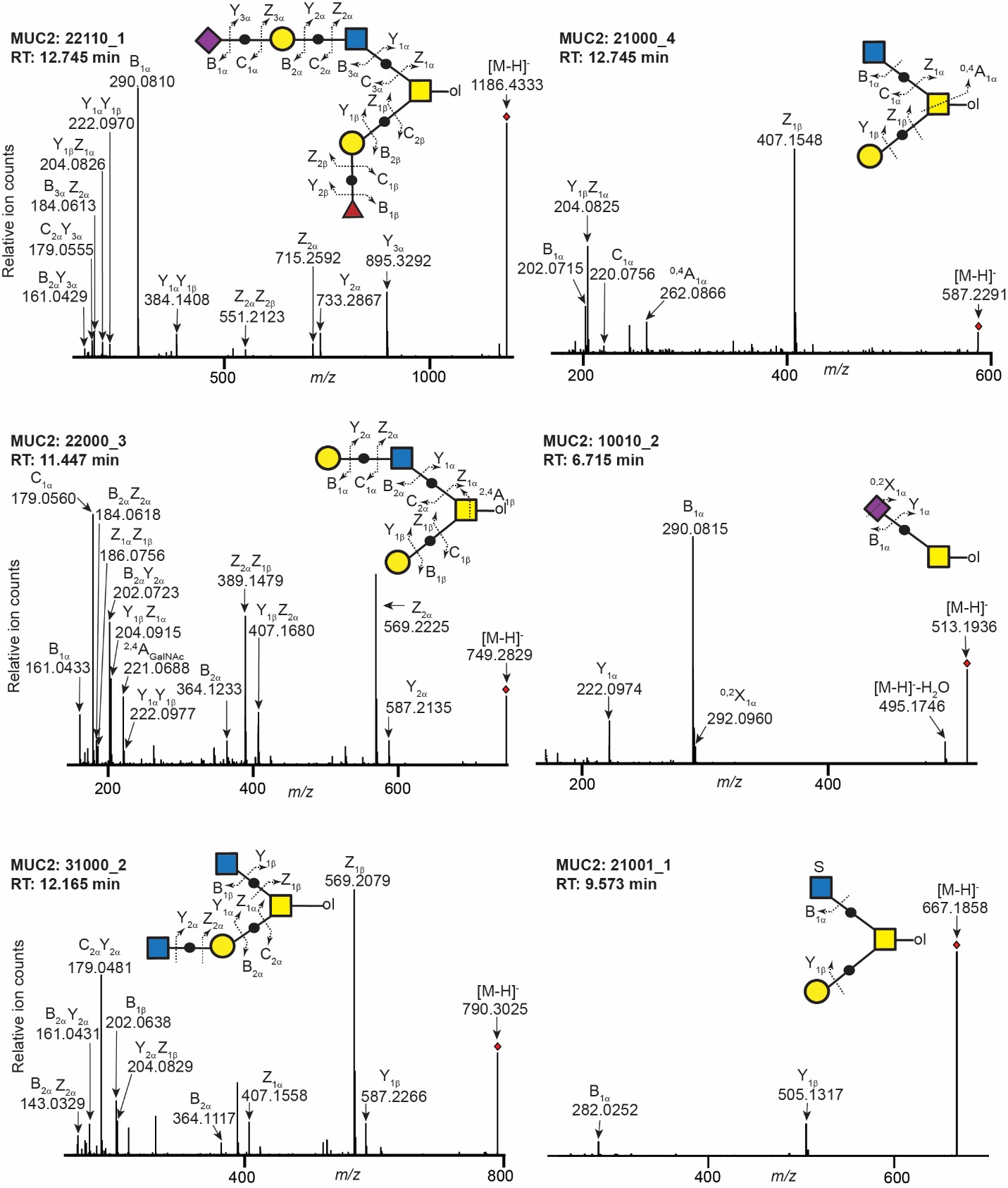


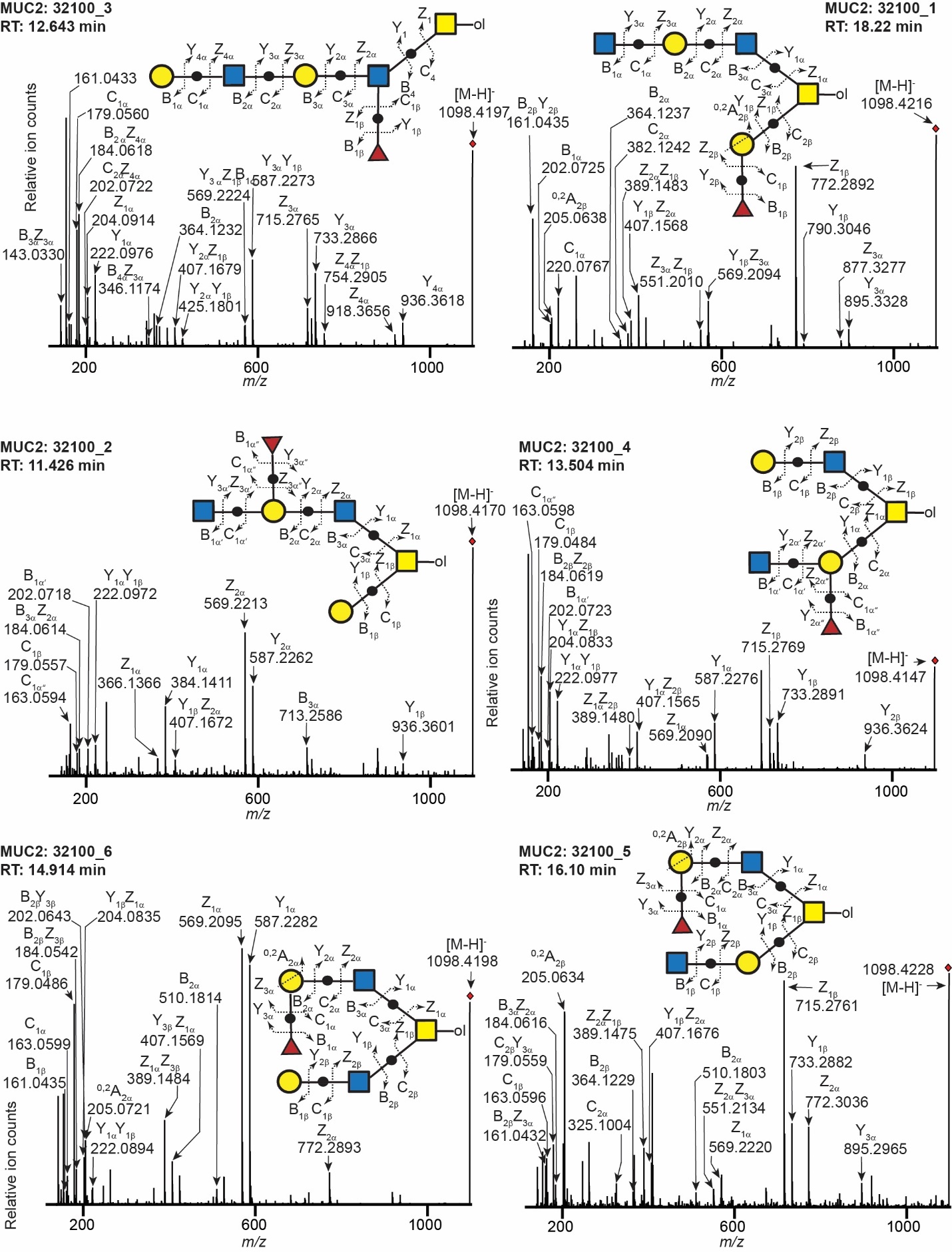


**Figure S10. Product ion spectra for selected infant-MUC2-derived *O*-glycans.**


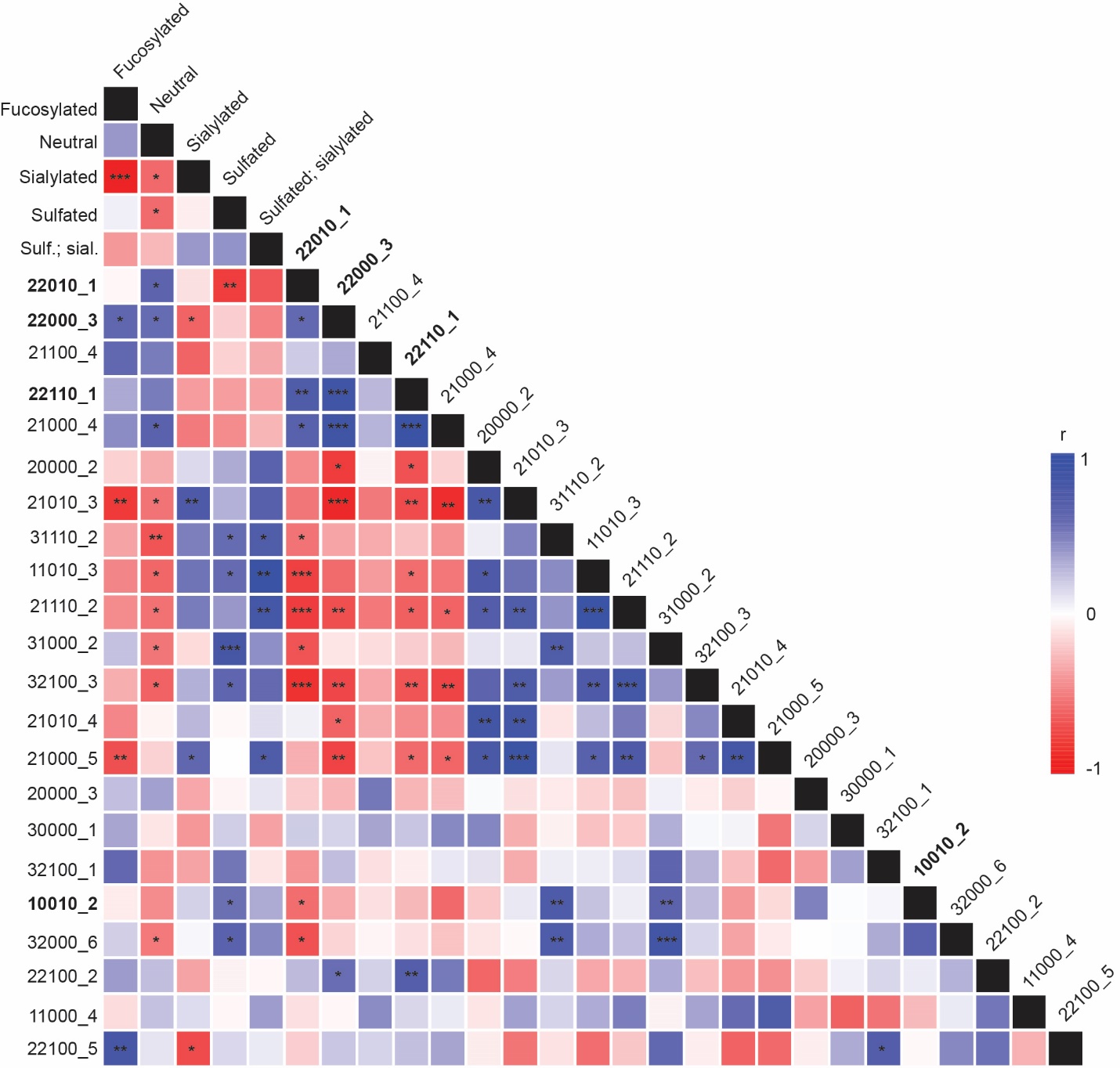


**Figure S11. Spearman’s correlation analysis reveals associations between the most abundant *O*-glycans detected across 15 neonatal MUC2 samples.** Spearman’s correlation coefficients were between the most abundant glycans or classes of glycans detected across *N* = 15 unique samples. Positive correlations are depicted in blue while negative correlations are in red; magnitude is indicated by color intensity and significant correlations are denoted *, **, or *** if *P* < 0.05, 0.01, or 0.05, respectively. As in Figure S2 and S7, glycans are represented by glyco-codes where each of the first five digits denotes the number of *m*/*z*-distinct HexNAc, Hex, Fuc, Neu5Ac, and sulfate residues, respectively and the _1, _2, *etc.* suffix denotes the number of HPLC-resolved isobars. In contrast with HMOs, all putative *O*-glycans were assumed to contain a reduced GalNAc (*i.e.* an alditol) at their reducing ends. Glycans labelled in bold font were those highlighted by PLD-DA.


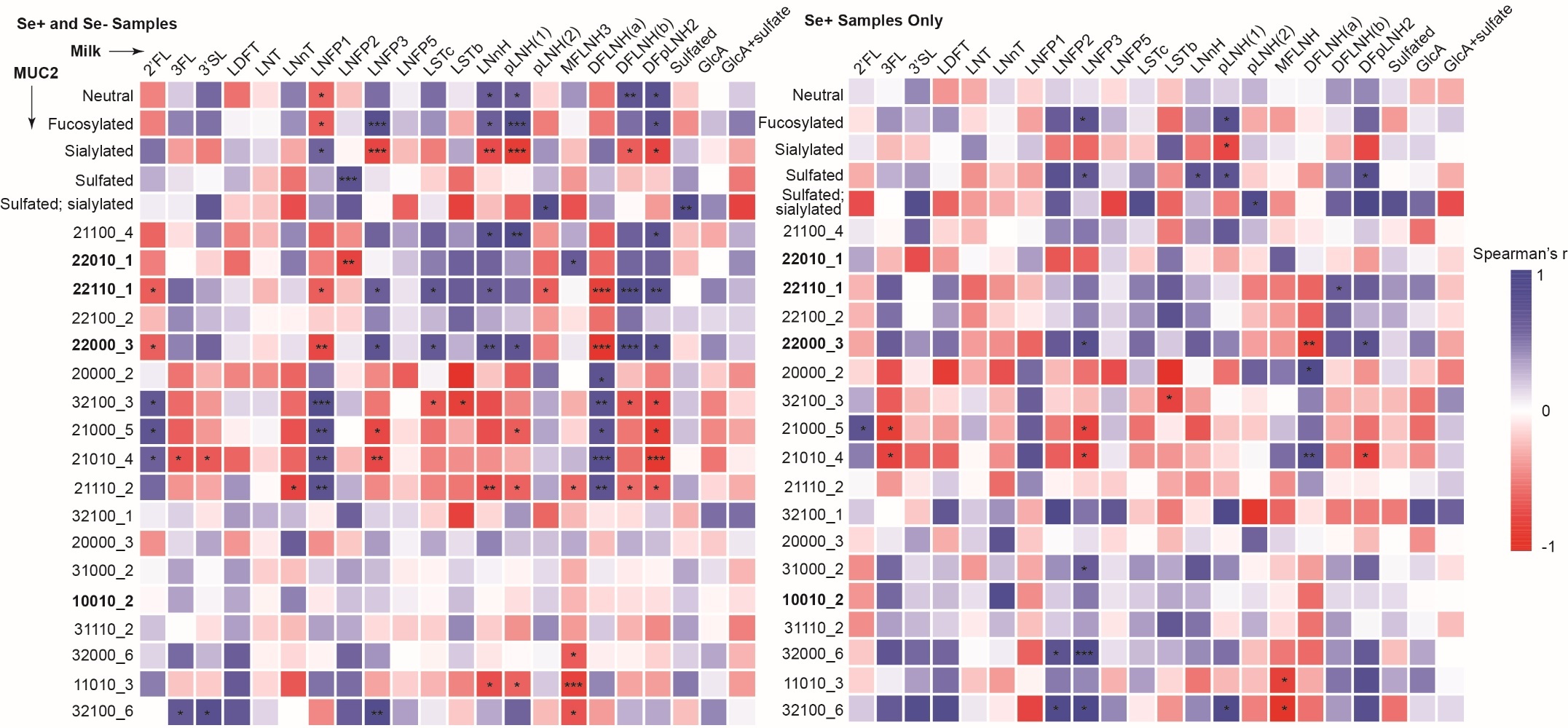


**Figure S12. Full heat maps depicting associations (Spearman’s r) between relative abundances of ingested HMOs and major MUC2 *O*-glycans.** HMO structures are depicted in Figure S2; the six-digit glyo-codes O-glycan codes are described above with those in bold font being identified most discriminating before the four sample groups by PLS-DA (Figure S8). Positive correlations are depicted in blue while negative correlations are in red; magnitude is indicated by color intensity and significant correlations are denoted *, **, or *** if *P* < 0.05, 0.01, or 0.05, respectively


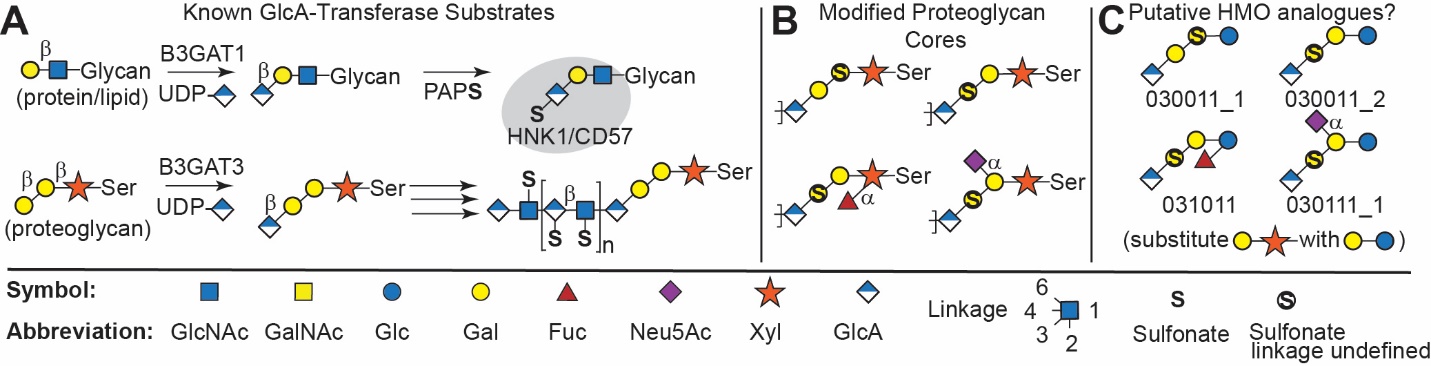


**Figure S13. Lactose-priming hypothesis: During lactation, high lactose and HMO concentrations divert GlcA away from proteoglycan biosynthesis and into HMOs. (A)** The HNK-1/CD57 glyco-epitope, expressed in the vertebrate brain and peripheral nervous tissues, consists of a 3-*O*-sulfonate-GlcA-β1,3-Gal-β1,4-GlcNAc-R trisaccharide wherein R is an *N*- or *O*-glycan (1) or glycolipid (2). A GlcA-transferase initially dubbed GlcAT-P (in rats (3)) was demonstrated to be necessary for the biosynthesis of neural HNK-1 in humans (4). GlcAT-P, commercially-available as β1,3-GlcA-transferase1 (B3GAT1), is reported to have somewhat conflicting acceptor scope, with some authors observing activity on both Gal-β1,3-Gal-β1,4-Glc-R and Gal-β1,4-Gal-β1,4-Glc-R (where R = a pyridylamine fluorophore) while others (5) noted activity on Gal-β1,4-GlcNAc but not the Gal-β1,3-GlcNAc congener. In human nervous tissues, a sulfo-transferase (NHK-1ST) completed HNK-1 biosynthesis upon the sulfonate transfer from 3’-phosphoadenosine-5’-phosphosulfate (PAPS) to the 3-OH group of the non-reducing GlcA residue (6). Although HNK-1ST does not appear active in other tissues, in mice a **B3GAT1** homologue was found to be expressed in the kidney wherein non-sulfated HNK1/CD57 was likewise detected (7). Although nothing, to the best of our knowledge, is currently known about the expression of these enzymes in the mammary gland, presuming that the same glycosyltransferases that produce lipid or protein-linked glycoconjugates also make HMOs, we initially hypothesized that bovine CD57-like MOs consisted of sulfonated GlcA-β1,3-Gal-β1,4-Glc analogues (8). Consistent with this hypothesis, ten MOs consisting of two hexoses (presumably Gal-β1,4-Glc, *i.e*. lactose), one GlcA and one sulfate were detected in bovine milk. Although in human milk, the most abundant GlcA/sulfate-containing HMO, 030011, has a composition theoretically consistent with CD57-like glycans, our HRMS and tandem MS data indicated the existence of GlcA/sulfate-containing HMOs also bearing Fuc and Neu5Ac residues, combinations of monosaccharide that, to our knowledge, represent novel glyco-epitopes. For example, our tandem MS (Figure 4A) suggests that HMO cores consisting of 2’FL and 3FL may both be elongated with GlcA and sulfate and that, in direct contrast with CD57, one of these contains an internal rather than non-reducing GlcA/sulfate. Jin and coworkers have also reported tandem MS evidence for two MOs bearing internal GlcA residues (in non-human samples) although no GlcA moieties described in this report were sulfated (9). Previously, GlcA-containing core-1 *O*-glycans have been detected in tissues collected from *Drosophila* embryos (10); these glycans contained both reducing and internal GlcA residues, none of which were sulfated. Meanwhile, more complex, branched core-1 or -2 *O*-glycans simultaneously bearing GlcA, Fuc, and sulfate residues have been detected as main acidic components of the mucosal coating of amphibian eggs; in these glycans, the sulfate moieties were borne only on GlcNAc and Gal residues, in contrast with CD57, while the GlcA moiety was β1,3-linked to Gal as it is in CD57 (11). More recently, in mammals, Vos *et al*. presented HRMS data for two core-1 *O*-glycans on bovine submaxillary mucin (12). However, in keeping with the idea that the 030011 series of glyco-codes resemble the linker tetrasaccharide common to GAGs like heparan or chondroitin, we note that the GlcA-β1,3-Gal-β1,3-Gal-β1,4-Xyl-β-*O*-Ser/Thr GAG core is completed by the enzyme β1,3-GlcA-transferase3 (**B3GAT3**, also known as GlcAT-I), an enzyme with strict specificity for the Gal-β1,3-Gal-R (13) in contrast with the broader scope of B3GAT1. **(B)** GAG linkers modified with sulfate, fucose, Neu5Ac and/or Neu5Gc residues (14–16) have previously been reported; a small subset of these is shown. Interestingly, HNK-1ST has previously been shown to sulfonate a GlcA residue in the context of a GAG linker (17) (not shown). **(C)** Since both lactose and HMOs exceed GAG biosynthesis (in terms of their percentage by mass) in the mature mammary gland (18), we hypothesize that lactose (Gal-β1,4-Glc) may mimic the Gal-β1,4-Xyl moiety of GAGs, thereby diverting GAG biosynthetic enzymes and creating Fuc-, Neu5Ac-, and sulfate-modified GAG linkers on a lactose core to generate a range of HMOs with the novel glyco-codes identified in the human milk and infant stool samples analyzed herein.


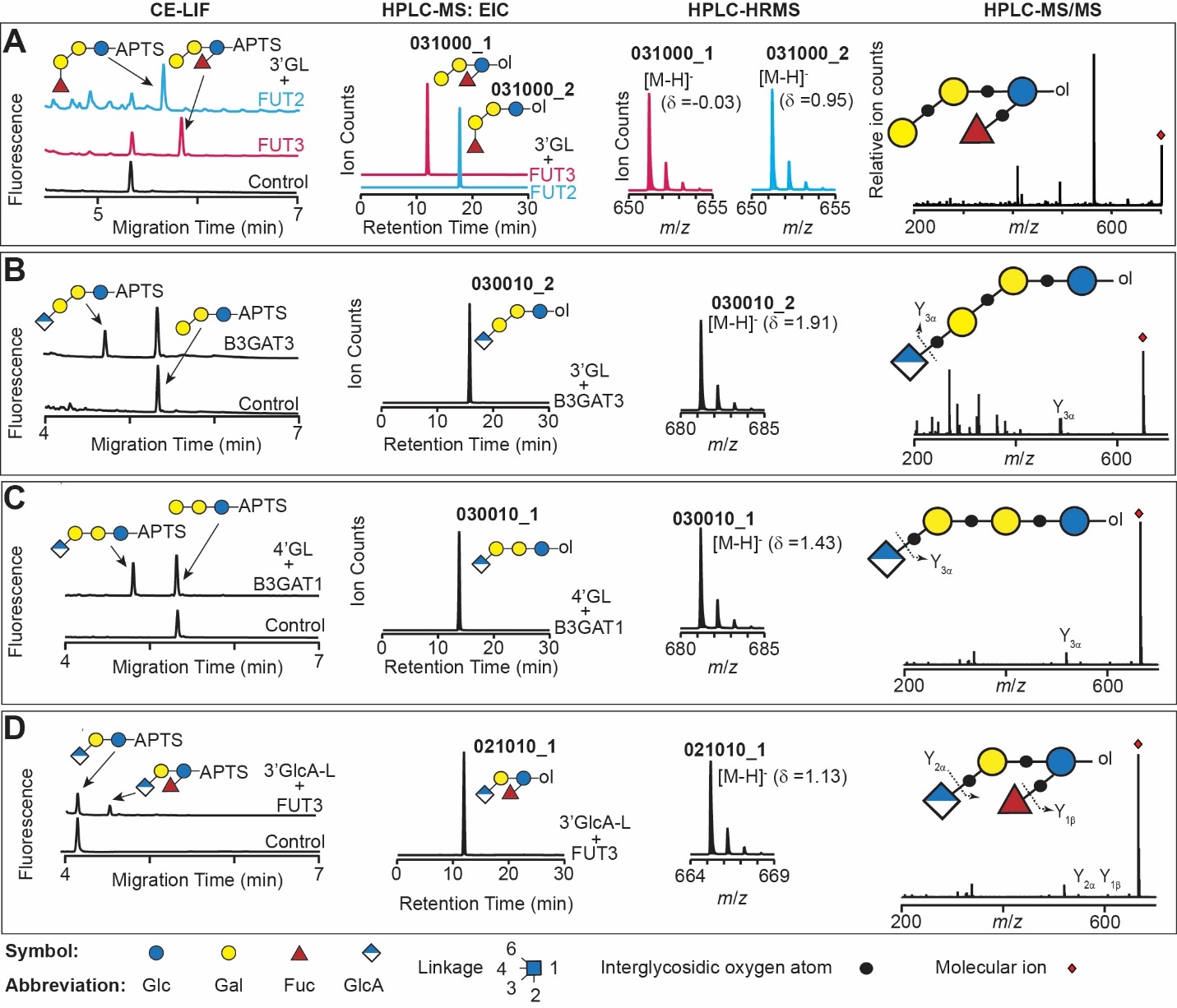


**Figure S14. Key CE-LIF and HPLC-MS evidence for the biosynthesis of lactose-primed GAG-like-linkers.** Commercial GlcA- and Fuc-transferases were used to enzymatically prepare novel GlcA- or Fuc-containing HMOs. For each test reaction, APTS-labelled products were resolved by CE-LIF without desalting while samples analyzed by HPLC-MS were dried, reduced to alditols (denoted by the “ol”) and desalted using PGC. For samples analyzed by HPLC, extracted ion chromatograms (EICs), a full scan, high resolution mass spectrum (with the mass error of the [M-H]^-^ molecular ion in ppm indicated), and a product ion scan are depicted. **(A)** 3β’-galactosyllactose (3’GL) and 4β’-galactosyllactose (4’GL) were tested as substrates for FUT2 and FUT3. 3’GL was accepted by both enzymes whereas 4’GL was only a substrate for FUT2 (not shown). **(B)** While both B3GAT3 and B3GAT1 accepted 3’GL as a substrate (only data from B3GAT3 are shown), **(C)** 4’GL was only a substrate for B3GAT1. Likewise, *N*-acetyllactosamine was also a substrate for B3GAT1 (not shown). Neither 3FL nor 2’FL were substrates for B3GAT1 or B3GAT3, however, **(D)** GlcA-β1,3-lactose (3’GlcA-L) was a substrate for FUT3. Therefore, since 3’GL was a substrate for both B3GAT3 and FUT3, it is hypothesized that Fuc- and GlcA-containing HMOs contain a 3’GL core bearing a Fuc residue on the reducing end Glc moiety of lactose, analogous to Fuc-modified proteoglycan cores.

**Supporting References:**

1. Morise, J., Kizuka, Y., Yabuno, K., Tonoyama, Y., Hashii, N., Kawasaki, N., Manya, H., Miyagoe-Suzuki, Y., Takeda, S., Endo, T., Maeda, N., Takematsu, H., and Oka, S. (2014) Structural and biochemical characterization of O-mannose-linked human natural killer-1 glycan expressed on phosphacan in developing mouse brains. *Glycobiology*. **24**, 314–324

2. Needham, L. K., and Schnaar, R. L. (1993) The HNK-1 reactive sulfoglucuronyl glycolipids are ligands for L-selectin and P-selectin but not E-selectin. *Proc. Natl. Acad. Sci. U. S. A.* **90**, 1359–1363

3. Terayama, K., Oka, S., Seiki, T., Miki, Y., Nakamura, A., Kozutsumi, Y., Takio, K., and Kawasaki, T. (1997) Cloning and functional expression of a novel glucuronyltransferase involved in the biosynthesis of the carbohydrate epitope HNK-1. *Proc. Natl. Acad. Sci. U. S. A.* **94**, 6093–6098

4. Shimoda, Y., Tajima, Y., Nagase, T., Harii, K., Osumi, N., and Sanai, Y. (1999) Cloning and expression of a novel galactoside β1,3- glucuronyltransferase involved in the biosynthesis of HNK-1 epitope. *J. Biol. Chem.* **274**, 17115–17122

5. Kakuda, S., Shiba, T., Ishiguro, M., Tagawa, H., Oka, S., Kajihara, Y., Kawasaki, T., Wakatsuki, S., and Kato, R. (2004) Structural basis for acceptor substrate recognition of a human glucuronyltransferase, GlcAT-P, an enzyme critical in the biosynthesis of the carbohydrate epitope HNK-1. *J. Biol. Chem.* **279**, 22693–22703

6. Ong, E., Yeh, J. C., Ding, Y., Hindsgaul, O., and Fukuda, M. (1998) Expression cloning of a human sulfotransferase that directs the synthesis of the HNK-1 glycan on the neural cell adhesion molecule and glycolipids. *J. Biol. Chem.* **273**, 5190–5195

7. Tagawa, H., Kizuka, Y., Ikeda, T., Itoh, S., Kawasaki, N., Kurihara, H., Onozato, M. L., Tojo, A., Sakai, T., Kawasaki, T., and Oka, S. (2005) A non-sulfated form of the HNK-1 carbohydrate is expressed in mouse kidney. *J. Biol. Chem.* **280**, 23876–23883

8. Gray, T. W., Narayana, K., Garner, A. M., Bakker, S. A., Yoo, R. K., Fischer-Tlustos, Amanda, J., Steele, M. A., and Zandberg, W. F. (2021) Analysis of the biosynthetic flux in bovine milk oligosaccharides reveals competition between sulfated and sialylated species and the existence of glucuronic acid-containing analogues. *Food Chem.* **361**, 130143

9. Jin, C., Lundstrøm, J., Korhonen, E., Luis, A. S., and Bojar, D. (2023) Breast Milk Oligosaccharides Contain Immunomodulatory Glucuronic Acid and LacdiNAc. *Mol. Cell. Proteomics*. **22**, 100635

10. Aoki, K., Porterfield, M., Lee, S. S., Dong, B., Nguyen, K., McGlamry, K. H., and Tiemeyer, M. (2008) The diversity of O-linked glycans expressed during Drosophila melanogaster development reflects stage- and tissue-specific requirements for cell signaling. *J. Biol. Chem.* **283**, 30385–30400

11. Mourad, R., Morelle, W., Neveu, A., and Strecker, G. (2001) Diversity of O-linked glycosylation patterns between species: Characterization of 25 carbohydrate chains from oviducal mucins of Rana ridibunda. *Eur. J. Biochem.* **268**, 1990–2003

12. Vos, G. M., Weber, J., Sweet, I. R., Hooijschuur, K. C., Sastre Toraño, J., and Boons, G. J. (2023) Oxidative Release of O-Glycans under Neutral Conditions for Analysis of Glycoconjugates Having Base-Sensitive Substituents. *Anal. Chem.* **95**, 8825–8833

13. Fondeur-Gelinotte, M., Lattard, V., Gulberti, S., Oriol, R., Mulliert, G., Coughtrie, M. W. H., Magdalou, J., Netter, P., Ouzzine, M., and Fournel-Gigleux, S. (2007) Molecular basis for acceptor substrate specificity of the human β1,3-glucuronosyltransferases GlcAT-I and GlcAT-P involved in glycosaminoglycan and HNK-1 carbohydrate epitope biosynthesis, respectively. *Glycobiology*. **17**, 857–867

14. Klein, J. A., Meng, L., and Zaia, J. (2018) Deep sequencing of complex proteoglycans: A novel strategy for high coverage and sitespecific identification of glycosaminoglycanlinked peptides. *Mol. Cell. Proteomics*. **17**, 1578–1590

15. Toledo, A. G., Nilsson, J., Noborn, F., Sihlbom, C., and Larson, G. (2015) Positive mode LC-MS/MS analysis of chondroitin sulfate modified glycopeptides derived from light and heavy chains of the human inter-α-trypsin inhibitor complex. *Mol. Cell. Proteomics*. **14**, 3118–3131

16. Nilsson, J., Noborn, F., Gomez Toledo, A., Nasir, W., Sihlbom, C., and Larson, G. (2017) Characterization of Glycan Structures of Chondroitin Sulfate-Glycopeptides Facilitated by Sodium Ion-Pairing and Positive Mode LC-MS/MS. *J. Am. Soc. Mass Spectrom.* **28**, 229–241

17. Hashiguchi, T., Mizumoto, S., Nishimura, Y., Tamura, J. I., Yamada, S., and Sugahara, K. (2011) Involvement of Human Natural Killer-1 (HNK-1) sulfotransferase in the biosynthesis of the GlcUA(3-O-sulfate)-Gal-Gal-Xyl tetrasaccharide found in α-thrombomodulin from human urine. *J. Biol. Chem.* **286**, 33003–33011

18. Greenwood, M., Murciano-Martinez, P., Berrington, J., Flitsch, S. L., Austin, S., and Stewart, C. (2024) Characterising glycosaminoglycans in human breastmilk and their potential role in infant health. *Microb. Cell*. **11**, 221–234
